# Circular RNAs orchestrating breast cancer hallmarks: bridging tumor biology and therapy resistance

**DOI:** 10.1007/s10142-025-01709-8

**Published:** 2025-10-13

**Authors:** Abdelhamid M. Abdelhamid, Mai Ahmed Shafei, Mariam Gamaleldin, Alaa Bassam Heikal, Wesam H. Khidr, Nabil Hazza Al-Saadi, Ganna Magdy Kandil, Mahmoud M. Omarn, Maha Alhelf

**Affiliations:** 1 Applied Biotechnology Program, School of Biotechnology, Nile University, Giza, Egypt; 2 Faculty of Biotechnology, October University for Modern Sciences and Arts, Giza, Egypt; 3 Medical Biochemistry and Molecular Biology Department, Faculty of Medicine, Cairo University, Cairo, Egypt

**Keywords:** Breast cancer, Drug resistance, Personalized medicine, Biomarkers, Non-coding RNAs (ncRNAs), CircRNAs

## Abstract

Breast cancer (BC) remains a leading cause of cancer-related mortality among women worldwide, with treatment resistance posing a significant clinical challenge. Circular RNAs (circRNAs), a class of non-coding RNAs, have gained increasing attention as key regulators of gene expression, influencing BC pathogenesis, progression, and therapeutic response. This review explores the mechanistic insights into circRNA functions in BC, focusing on their involvement in tumor proliferation, metabolic reprogramming, epithelial-mesenchymal transition (EMT), angiogenesis, metastasis, and apoptosis. Additionally, we highlight the crosstalk between circRNAs and microRNAs, emphasizing their potential as diagnostic and prognostic biomarkers. Beyond their roles in tumor biology, circRNAs are implicated in drug resistance, modulating responses to chemotherapy, targeted therapy, and endocrine treatment. Despite their promising applications, challenges remain, including the complexity of circRNA interactions, and the development of robust preclinical models. Addressing these challenges through interdisciplinary research integrating genomics, transcriptomics, and functional studies will pave the way for circRNA-based therapeutic strategies and personalized medicine approaches in BC management.

## Introduction

Breast cancer (BC) was the second most commonly diagnosed cancer among women worldwide in 2022, according to GLOBOCAN 2022, with an estimated 2.3 million new cases, representing 11.6% of all global cancer cases. Moreover, it was the fourth leading cause of cancer-related mortality among women, accounting for approximately 666,000 deaths that year (Abdelhamid et al. 2024; Bray et al. 2024). Despite advances in early diagnosis and treatment, challenges persist, particularly in advanced stages where 5-year survival rates are alarmingly low at 26% mainly due to metastatic disease and resistance to therapy (Malmgren et al. 2018). Multiple factors including female sex, advanced age, family history, and genetic mutations influence BC risk (Łukasiewicz et al. 2021). These challenges highlight the need to develop personalized treatments and reduce reliance on chemotherapy by identifying novel therapeutic targets and biomarkers.

Whole-genome transcriptome analyses have revealed that around 80% of the human genome is transcribed, Yet only 2% encodes proteins, leaving over 90% transcribed into non-coding RNAs (ncRNAs), which do not directly govern protein synthesis (Palazzo and Koonin 2020). Subsequently, ncRNAs have been systematically classified into housekeeping and regulatory groups. Housekeeping ncRNAs consist of tRNAs and rRNAs, small nuclear RNAs (snRNAs) and small nucleolar RNAs (snoRNAs). Regulatory ncRNAs are categorized by their transcript length into short ncRNAs (< 200 nucleotides), such as microRNAs (miRNAs), PIWI-interacting RNAs (piRNAs), small interfering RNAs (siRNAs), and small nucleolar RNAs (snoRNAs), and long ncRNAs (lncRNAs) (> 200 nucleotides). These ncRNAs play crucial roles in RNA maturation, processing, signaling, gene expression, and protein synthesis (Doghish et al. 2025; Rostom et al. 2025).

In tumorigenesis, ncRNAs serve various roles; they can function as tumor suppressors in one type of cancer while facilitating tumor progression in another (Abdelhamid et al. 2024; Assal et al. 2025; Bhan et al. 2017; Doghish et al. 2025; Rostom et al. 2025). Notably, a new subclass of lncRNAs, known as circular RNAs (circRNAs), has garnered significant interest due to their emerging therapeutic potential, high stability, diagnostic and prognostic value and their critical involvement in cellular proliferation and malignant transformation processes (Meng et al. 2017).

CircRNAs represent a distinct class of endogenous non-coding RNAs characterized by a covalently closed loop structure, lacking the 5′−3′ polarity and poly-A tail, providing high stability against exonucleases (Meng et al. 2017). While their biogenesis can occur through various mechanisms, backsplicing is the predominant process in humans. Initially considered as mere byproducts of pre-mRNA splicing, circRNAs have since been recognized for their significant roles in regulating gene expression (Verduci et al. 2021). They play a crucial role in serving as microRNA sponges, protein scaffolds, and modulators of mRNA synthesis, splicing, and protein production (Liu et al. 2022). CircRNAs have been found to be associated with many biological processes such as gene expression regulation, protein interaction, encoding for proteins and peptides that inhibit cancer development, in addition to being involved in many physiological functions (Shi et al. 2023).

Research into underlying mechanisms has unveiled the pivotal roles of circRNAs in BC, influencing processes such as cell proliferation, apoptosis, angiogenesis, epithelial to mesenchymal transition (EMT), tumor microenvironment, and drug resistance. Moreover, emerging research into circRNAs in cancer has unraveled their potential as therapeutic targets (Hussen et al. 2023; Kumar et al. 2022). CircRNAs, whether functioning as oncogenes or tumor suppressors, may be harnessed as treatment targets. The unique back-splicing junction sequence of circRNAs allows for precise targeting of these molecules without interference with the corresponding parental mRNA, offering a promising avenue for therapeutic intervention (Beilerli et al., 2022). Despite these findings, the reasons behind the abnormal expression of circRNAs and the regulatory mechanisms governing circRNAs in tumors are still not fully understood (Zeng et al. 2022; H. da Zhang et al. 2018). In this review, we explore the role of circRNAs in BC, focusing on their biogenesis, dysregulation, and interactions with key signaling pathways. Additionally, we examine the therapeutic potential of circRNAs in overcoming drug resistance and their utility as diagnostic and prognostic biomarkers.

## Classification of circRNAs

CircRNAs are a type of RNA with a covalently closed loop structure, making them more stable than linear RNA (Mumtaz et al. 2020). CircRNAs initially could not be differentiated based on their size and their lack of 5’- and 3’ ends as well as poly(A) tails made it difficult to identify them by traditional sequencing approaches. However, the advancement in high throughput sequencing methods have allowed for better classification of these circRNAs based on their origin which are derived from specific protein coding regions in the genome (Jeck and Sharpless 2014).

These circRNAs can be classified into 3 types (J. Li et al. 2023), the first type is known as exonic circular RNAs (ecircRNA) which have been found to be derived from the coding region of genes and are predominately localized in the cytoplasm, their primary role is to regulate gene expression following transcription. The second type, intronic circular RNAs (ciRNAs) which originate from intronic regions of genes, the region of genes that do not code for proteins, are mostly located in the nucleus, they actively participate in the coordination of the complex transcription process (Zhang et al. 2013). The third type are known as exon-intron circular RNAs (EIciRNAs), these circRNAs can form a composition with exons which interact with RNA polymerase II enabling gene expression coordination (Li et al. 2015; Zhang et al. 2013).

## CircRNAs nomenclature

With the evolving field of research on circRNAs, the absence of standardized nomenclature has been a significant challenge for researchers (Liu et al. 2019; Youness et al. 2024). Current databases, including circBase 0.1 (http://www.circbase.org/), use arbitrary numbering and have limited information regarding the host gene and chromosomal locations of certain circRNAs (Youness et al. 2024). Consequently, a new nomenclature system based on the host gene and exact start/end positions within that gene has been developed to solve these issues and to facilitate clear communication between researchers (Moghaddam et al. 2022). The circBank database not only organizes circRNA data comprehensively but also provides detailed knowledge on miRNA binding sites, conservation, circRNA mutations, m6A modifications, protein-coding potential, and predicted internal ribosome entry sites (IRESs), offering a basis for further development of circRNA nomenclature and functions (Youness et al. 2024).

**Fig. 1:**
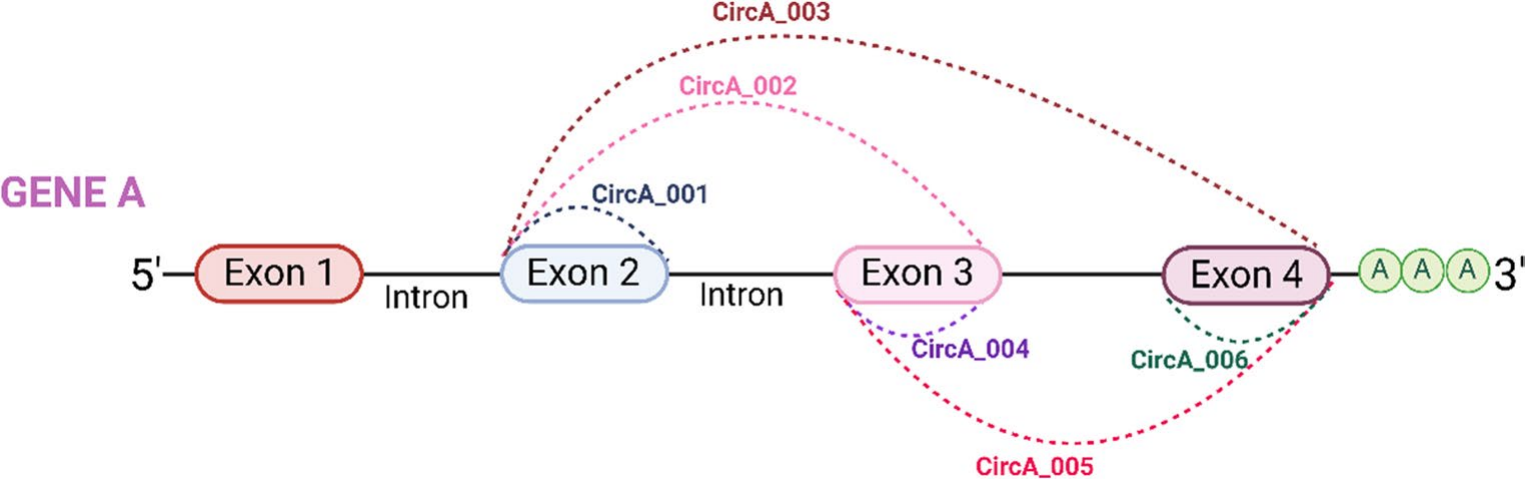
Nomenclature of circRNAs in CircBank. Schematic representation of alternative back-splicing events generating multiple circRNA isoforms from GENE A. Exons are represented as colored boxes, and introns as connecting lines. The arcs represent back-splicing junctions. CircA_001 to CircA_006 indicate specific circRNA isoforms: CircA_001: circularization of Exon 2, CircA_002: Exons 2–3, CircA_003: Exons 2–4, CircA_004: Exon 3, CircA_005: Exons 3–4, and CircA_006: Exon 4. Each circRNA is formed by a back-splicing event that Links a downstream 5′ splice donor site to an upstream 3′ splice acceptor site

Human circRNAs are named based on the human genome organization (HUGO) host gene symbol, following this format: “hsa-circHUGO-#”, according to the circBank (Seal et al. 2020). Additionally, circRNAs originating from the same host genes are numbered based on their position within the gene, with the upstream one given the initial number. If circRNAs have the same starting point but different ending point, the one with the earlier ending point gets the lower number (see Fig. 1). For circRNAs with identical starting and ending points, alternative splicing is taken into account. In this regard, the circRNAs nomenclature includes “has-circHUGO-#_V#”, where “V” represents “variant”, and the number following “V” is determined by the length of the circRNA (Mumtaz et al. 2020; Seal et al. 2020).

For the intergenic circRNAs’ nomenclature, the format “has-circChrom#_#” is applied, where the first number represents the chromosome number, and the circRNA order number is assigned according to the same rules as those for circRNAs derived from coding genes (Moghaddam et al. 2022).

## CircRNAs biogenesis

The biogenesis of most circRNAs occurs through a co-transcriptional, spliceosome-mediated process known as back-splicing. In this process, a 5′ splice donor site covalently Links to an upstream 3′ splice acceptor site in the associated linear RNA, resulting in the circularization of the intervening exon(s) (Nielsen et al. 2022). Consequently, the formation of circRNA hinders the synthesis of mRNAs originating from the same genomic locus. Requirements for the splicing and circularization of exons have been identified by various groups, whereby circularization signals reside within the introns flanking circle forming exons where protein factors bind to flanked intron sequences (Patop et al. 2019).

**Fig. 2:**
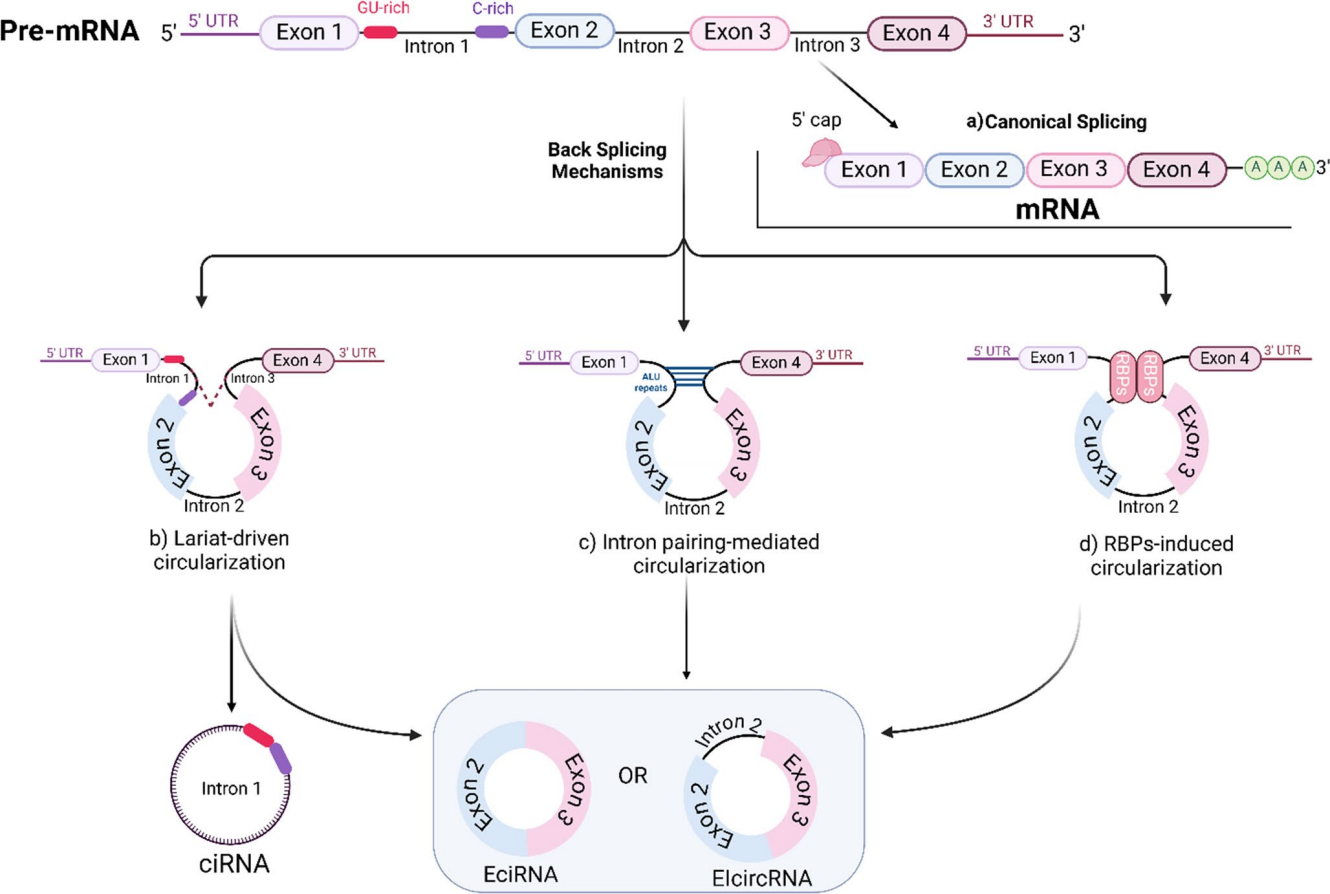
Biogenesis of circRNAs. Pre-mRNA can follow two main processing pathways: (**a**) canonical splicing, which results in linear mRNA, or (**b**–**d**) alternative back-splicing mechanisms that generate circRNAs. (**b**) Lariat-driven circularization leads to the formation of an exon-containing lariat During exon-skipping events, producing eIciRNA or ecircRNA. In contrast, ciRNAs are derived from intron lariats that evade debranching following canonical splicing, with a distinctive 7-nucleotide GU-rich motif adjacent to the 5′ splice site and an 11-nucleotide C-rich motif near the branch point. (**c**) Intron pairing-mediated circularization occurs through complementary base-pairing between flanking intronic sequences, such as ALU repeats. (**d**) RBP-induced circularization is facilitated by RNA-binding proteins (RBPs) that bring splice sites into proximity, promoting circRNA formation

In eukaryotes, pre-mRNAs undergo two distinct splicing processes: “canonical splicing,” which generates linear mRNAs by splicing introns and joining exons, and “non-canonical splicing” or “back splicing,” which produces circRNAs by forming stable, covalently closed-loop structures without 5′ caps or 3′ poly(A) tails (Dawoud et al. 2024; J. Li et al. 2023). CircRNA loop formation involves junctions that are specific to each circRNA. Understanding these mechanisms is pivotal to understanding circRNA functions and enhancing their detection and analysis (Huang et al. 2020). Currently, two widely accepted circularization mechanisms explain this, as illustrated in Fig. 2.

### Lariat-driven circularization

In this circularization model, the pre-mRNA undergoes partial folding in a way that brings the 5′ splice donor (SD) site of a downstream exon into proximity with the 3′ splice acceptor (SA) site of an upstream exon (He et al. 2021a, b). This interaction facilitates exon skipping, leading to the formation of a lariat structure that encapsulates both exons and introns. The resulting lariat, which includes the skipped exon, subsequently undergoes internal splicing to produce ecircRNA through back-splicing (Y. Huang and Zhu 2021). Introns can also be removed by the action of the lariat debranching enzyme (DBR), which linearizes intron lariats for degradation (Carriero and Damha 2003). However, if the introns are not fully removed during this process, an exon-intron circRNA (EIcircRNA) will be produced instead (Dawoud et al. 2023).

Mechanistic evidence suggests that the generation of a large lariat encompassing the skipped exon is a prevalent and crucial stage in circRNA biogenesis, particularly during indirect back-splicing processes (Sharpless and Norman 2014; Xiao et al. 2018) lariat, produced by exon skipping, acts as a precursor that undergoes back-splicing, resulting in a mature circRNA molecule (Jiang et al. 2021; Li et al. 2019).

### Direct Back-splicing

In this method, if back-splicing occurs initially, the RNA precursor will directly make a circRNA along with an intermediate that contains both introns and exons. This intermediate is then further processed to produce a linear RNA (Robic and Kühn 2020). The method of “direct back-splicing” can be further classified into two groups based on the differences of the circularization mechanism: “Intron-pairing Driven Circularization” and " RNA-binding proteins (RBP)- Mediated Circularization (Y. Huang and Zhu 2021).

#### Intron-pairing driven circularization

Intron-pairing Driven Circularization is triggered by intronic flanks adjacent to circularized exons. A critical element in this process is a conserved motif found in both the upstream and downstream introns, identified as the canonical ALU repeat. Notably, these distinct characteristics of exon and intron length are strongly associated with the pr oduction of EIcircRNA, where introns are conserved, and ecircRNA, where introns are excised. This observation suggests that the circularization process is highly specific (He et al. 2021a, b).

#### RNA-binding proteins (RBP)-induced circularization

The relationship between RNA-binding proteins (RBPs) and circRNAs is bidirectional and context-dependent, with circRNAs also exerting regulatory control over RBP expression (Jiang et al. 2021). Here, the biogenesis of circRNAs is modulated by RBPs through various mechanisms discussed below (Zang et al. 2020). These proteins play a pivotal role in both promoting and inhibiting the formation of circRNAs, thereby significantly impacting their abundance and functional roles in different cellular contexts. The relationship between RBPs and circRNAs is bidirectional and context-dependent, with circRNAs also exerting regulatory control over RBP expression (Jiang et al. 2021).

One of the primary mechanisms through which RBPs facilitate circRNA formation is by the stabilization of the transient RNA structures necessary for back-splicing (Chen 2020). Double-stranded RNA-binding proteins (dsRBPs) such as NF90/NF110 have been demonstrated to enhance circRNA production by binding to intronic RNA sequences that flank circularized exons (Ren et al. 2022). NF90/NF110, through their dsRNA binding domains (dsRBDs), interact with inverted Alu repeats (IRAlus) within nascent pre-mRNA, thereby stabilizing the back-splicing event and promoting circRNA formation. This stabilization is crucial, as it ensures the correct alignment and pairing of complementary sequences, which are essential for the circularization process (Li et al. 2018).

Conversely, RBPs could also work as inhibitors by destabilizing the RNA duplexes required for back-splicing. For instance, the RNA helicase DHX9, which incorporates both a dsRBD and an RNA helicase domain, has been shown to play a significant role in the regulation of circRNA production by unwinding RNA duplexes that are essential for the back-splicing process, thus preventing the formation of circRNAs (Jiang et al. 2021). Adenosine deaminase acting on RNA 1 (ADAR1) inhibits circRNA production by editing adenosines to inosines in RNA duplexes, reducing sequence complementarity and destabilizing the RNA structure, which impairs circRNA formation. Interestingly, there appears to be a functional interplay between DHX9 and ADAR1 in the regulation of circRNA biogenesis, potentially due to their shared association with Alu elements (Rybak-Wolf et al. 2014).

## Mechanistic insights into circRNAs’ role in breast cancer

As previously discussed circRNAs are known for their stability, interact with RBPs and also engage in key growth signaling pathways such as MAPK/ERK and PTEN/PI3K/AKT. CircRNAs engage with RBPs through mechanisms including acting as protein sponges, decoys, scaffolds, or recruiters, thereby influencing the fate of their target mRNAs (Table 1). Additionally, some circRNAs possess an Internal Ribosome Entry site (IRES), enabling them to directly encode proteins (Lei et al. 2020). While circRNAs primarily function as miRNA sponges, their secondary function involves circRNA-protein interactions. Among the proteins that interact with RNA molecules, RBPs are particularly significant. These proteins play a crucial role in RNA metabolism, including processes such as maturation, transport, localization, and translation of RNAs, and are also involved in the formation of ribonucleoprotein complexes (Ceci et al. 2021). Therefore, circRNAs function through various mechanisms, including sponging miRNAs and interfering with RNA splicing, making them potential biomarkers and therapeutic targets for management of diseases including cancer (Ghazimoradi and Babashah 2022).

### CircRNAs as miRNA sponges

**Fig. 3:**
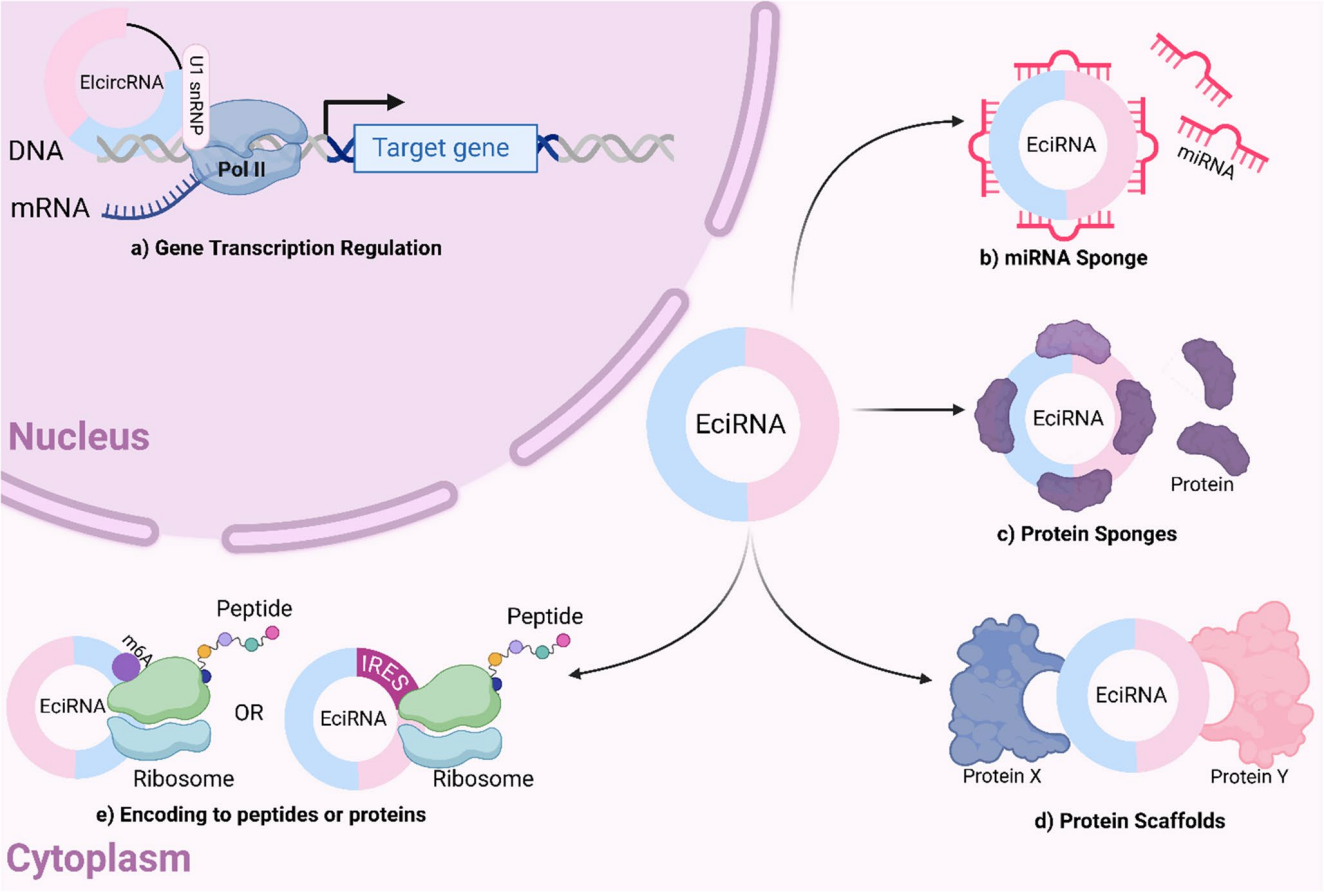
Biological Functions of circRNAs. CircRNAs exhibit multifaceted roles in both the nucleus and cytoplasm. (**a**) ElciRNAs enhance the transcription of parental genes by interacting with U1 snRNP and Pol II. (**b**) CircRNAs regulate miRNA activity by containing miRNA response elements (MREs), which sequester miRNAs and lead to either translational repression or mRNA degradation. (**c**) Some circRNAs possess internal ribosome entry sites (IRES) or undergo m6A modifications, allowing them to act as templates for peptide or protein synthesis. (**d**) CircRNAs serve as protein scaffolds, promoting the assembly of molecular complexes. (**e**) They also function as sponges for RNA-binding proteins (RBPs), thereby influencing gene expression

CircRNAs containing multiple miRNA binding sites can function as competing endogenous RNAs (ceRNAs) by binding to miRNAs via miRNA response elements (MREs), thereby inhibiting their activity (Fig. 3) (Singh et al. 2024). For example, a recent study assessing autophagy-associated circRNA circCDYL found that circCDYL promoted autophagic level in BC cells via the miR-1275-ATG7/ULK and may serve as a potential prognostic and predictive marker for BC patients (Liang et al. 2020).

CircIFI30 promotes the progression of triple-negative breast cancer (TNBC) and may function as a ceRNA for miR-520b-3p, counteracting its suppressive impact on the *CD44* gene, thereby promoting the progression of TNBC (Xing et al. 2020). Therefore, the circIFI30/miR-520b-3p/CD44 axis could serve as a novel diagnostic and prognostic marker, as well as a potential therapeutic target for TNBC patients (Xing et al. 2020). Circ_0006528 contributes to paclitaxel (PTX) resistance in BC by sponging miR-1299, thereby upregulating cyclin-dependent kinase 8 (CDK8) expression, which is linked to poor patient prognosis (Xing et al. 2020). Inhibition of Circ_0006528 in paclitaxel-resistant BC cells disrupts EMT and enhances tumor cell clearance, while promoting apoptosis and impeding tumor growth, underscoring its potential as a therapeutic target in overcoming drug resistance (Wang et al. 2023a, b) (Table 1).

### CircRNAs as protein sponges

CircRNAs can act as protein sponges or decoys, affecting cellular functions by modulating protein expression and function, while also influencing their own synthesis and degradation through RNA-protein interactions (Fig. 3) (Huang et al. 2020). RBPs are the most common class of proteins which interact with RNA molecules. These proteins play a crucial role in RNA metabolism, including processes such as maturation, transport, localization, and translation of RNAs, influencing their roles in BC (Conlon and Manley 2017). Similarly, circSKA3 enhances tumor progression as it promotes migration and invasion of BC cells and tissues by forming a complex with Tks5 and integrin β1. This results in the formation of invadopodium which are responsible for cancer invasiveness and metastasis (Du et al. 2020).

Other studies demonstrated circ-Dnmt1 oncogenic behavior in BC, which promotes tumor growth by inhibiting cellular senescence through autophagy modulation. It achieves this by facilitating the nuclear translocation of p53 and AUF1, which interact with circ-Dnmt1 to enhance cell proliferation and survival (Du et al. 2018). CircAMOTL1, a newly identified circRNA, enhances c-Myc nuclear translocation in BC and is linked to increased cell viability, invasion, and Paclitaxel (PTX) resistance. In BC tissues, circAMOTL1 is overexpressed, resulting in a significant increase in both phosphorylated and total AKT protein (Sadlak et al. 2023). Additionally, circAMOTL1 modulates the gene and protein expression of AKT-related pro-apoptotic (BAX and BAK) and anti-apoptotic (BCL-2) factors, indicating its potential role in stabilizing AKT signaling. These results suggest the important role circAMOTL1 may play in the Paclitaxel (PTX) resistance of BC cells. Knockdown of circAMOTL1 disrupts these processes, highlighting its potential as a therapeutic target for BC treatment (Sun et al. 2020) (Table 1).

### CircRNAs as molecular scaffolds

CircRNAs can also function as scaffolds facilitating contact between two or more proteins (Fig. 3) (Zhou et al. 2020a, b). Circ-Ccnb1 functions as a scaffold that modulates interactions between key proteins depending on the p53 mutation status (Almouh et al. 2022). In wild-type p53 cells, circ-Ccnb1 facilitates the interaction between p53 and H2AX, promoting cell survival. However, in p53 mutant cells, circ-Ccnb1 forms a complex with H2AX and Bclaf1, leading to cell death. These findings suggest that Ccnb1 scaffolding could be targeted to develop new therapeutic strategies against p53 mutations (Fang et al. 2018).

Notably, a recent study identified that circEIF3H interacts with the RNA-binding proteins IGF2BP2 and HuR, both of which are known to stabilize mRNAs. Through this interaction, three target genes—HSPD1, RBM8A, and G3BP1 have been identified (Yang et al. 2024). HSPD1 (also known as HSP60) is crucial for protein folding and mitochondrial assembly, and is secreted by cancer cells, contributing to processes such as transformation, angiogenesis, and metastasis (Javid et al. 2022). RBM8A, while associated with poor prognosis and tumor progression in hepatocellular carcinoma has an undetermined role in BC (R. Liang et al. 2021a, b). G3BP1, on the other hand, is known to enhance tumor cell proliferation and metastasis while inhibiting apoptosis through pathways involving Ras, TGF-β/Smad, Src/FAK, and p53. In BC, G3BP1 drives cell proliferation through the upregulation of PMP22 expression (Song et al. 2022). Together, the oncogenic activity of these genes’ underscores circEIF3H’s pivotal role in cancer progression and its potential as a target for personalized therapy in TNBC (Table 1) (Song et al. 2022).

### CircRNAs’ translational roles

CircRNAs were once presumed to be untranslatable. However, studies have shown that circRNAs possess open reading frame (ORF) features, along with IRES or m6A modifications that can be effectively translated into long proteins in eukaryotic translation systems through a rolling circle amplification (RCA) mechanism potential for circRNA translation in human cells (Fig. 3) (Chen et al. 2021). For instance, circFBXW7, a circRNA derived from the FBXW7 Gene encodes a 185-amino acid peptide (FBXW7-185aa) with tumor-suppressive properties. This peptide competitively interacts with USP28, counteracting USP28-induced c-Myc stabilization. Notably, the upregulation of FBXW7-185aa has been shown to inhibit the proliferation and migration of TNBC cells by increasing FBXW7 levels and promoting c-Myc degradation (Ye et al. 2019). FBXW7 was found to be downregulated in TNBC therefore upregulation of this circRNA may be used therapeutically (Table 1).

Additionally, increased expression of circ-EIF6 was found to promote cell proliferation and metastasis in TNBC in vivo and in vitro (Li et al. 2022). This circRNA which encodes the novel peptide EIF6-224 amino acid (aa), contains a 675-nucleotide (nt) open reading frame (ORF) with an IRES required for translation initiation. Expression of EIF6-224aa led to increased cell proliferation and migration mediated by inhibition of the ubiquitin-proteasome pathway and subsequent activation of the Wnt/beta-catenin pathway (Li et al. 2022). Similarly, circSEMA4B has been demonstrated to encode SEMA4B-211aa (Wang et al. 2022). However, mechanistically, SEMA4B-211aa inhibited the generation of PIP3 by competing with p110 to bind to p85, thereby inhibiting the phosphorylation of AKT (Thr308) leading to reduced activation of the PI3K/Akt pathway and inhibition of cell proliferation. Therefore, restoration of circSEMA4B in BC patients with low expression levels may have therapeutical potential (Wang et al. 2022) (Table 1).

### CircRNAs as transcription regulators

CircRNAs are known to modulate the transcription of their host genes either positively or negatively by interacting in a cis acting manner with RNA polymerase II (Pol II) in promotor regions, recruiting various proteins, or forming an R-loop that targets transcriptional regulatory regions of the host genes (García-Muse and Aguilera 2019). Comprehensive research has demonstrated that most EIciRNAs predominantly localize in the nucleus, where they engage with RNA Pol II to modulate host gene transcription in a cis-acting manner, whereas the majority of the ecircRNAs are found to localize in the cytoplasm (Fig. 3) (Wei et al. 2023).

Nuclear run-on assays demonstrated that silencing circEIF3J and circPAIP2 reduced the transcription of their respective parental genes, EIF3J and PAIP2, whereas direct silencing of these genes via siRNA did not significantly affect their transcription. RNA-DNA double FISH confirmed the co-localization of circEIF3J and circPAIP2 with their parental gene loci, suggesting that these circRNAs may regulate their gene expression in a cis-acting manner (Bose and Ain 2018). CircRNAs could also regulate transcriptional gene expression by serving as miRNA sponges as represented in Table 1.

**Table 1:** Mechanistic insight into circRNA roles in breast cancer

CircRNA	Functional role	Interaction	Effect	References
circFOXO3	Protein scaffold	p53/p21-CDK2	Inhibits cell proliferation and induces apoptosis	(Du et al. 2017)
circ-0011946	miRNA sponge	miR-145-5p/RFC3	Promotes cell proliferation and migration	(Zhou et al. 2018)
circ-NR3C2	Protein scaffold	NR3C2	Promotes cell survival and resistance to apoptosis	(Fan et al. 2021)
circ-ABCB10	miRNA sponge	miR-1271/ABCB10	Promotes drug resistance and cell survival	(Liang et al. 2017)
circ-DNMT1	miRNA sponge	miR-1236-3p/DNMT1	Inhibits cellular senescence and promotes tumor growth	(Du et al. 2018)
circ-CNOT2	Protein scaffold	CNOT2	Enhances cell proliferation and survival	(Smid et al. 2019)
circAHNAK1	miRNA sponge	miR-421/RASA1	Cell proliferation.	(Xiao et al. 2019)
circ-tada2a-E6	miRNA sponge	miR-203a-3p/TADA2A	Promotes tumor growth and progression	(Xu et al. 2019)
FECR1	Protein recruiter	TET1	Induce invasion, metastasis	(Chen et al. 2018a, b)
circ-Ccnb1	protein scaffold	H2AX, p53; H2AX, Bcla1	Decrease Proliferation, induce apoptosis	(Fang et al. 2018)
circ-IRAK3	miRNA sponge	miR-942/IRAK3	Promotes inflammation and cancer progression	(He et al. 2017)
circ-gfra1	miRNA sponge	miR-34a/GFRA1/TLR4	Enhances cell survival and proliferation and paclitaxel resistance	(He et al. 2017)
circDNMT1	Protein translocation	p53, AUF1	Autophagy mediated cell proliferation, survival, and tumor growth	(Du et al. 2018)
circBACH2	miRNA sponge	miR-186-5p/miR-548c 3p/CXCR4	Cell proliferation, invasion, and metastasis.	(Wang et al. 2021a, b)
Circ-IKBKB	Protein decoy	Decoys NF-κB pathway proteins	Promotes inflammation and cancer progression	(Xu et al. 2021)
circ-PDCD11	miRNA sponge	miR-512-3p/CDCA3/PDCD11	Increased glucose uptake, lactate production, ATP generation, Promote tumorigenesis.	(Xing et al. 2021)
circMTO1	Protein scaffold	TRAF4/Eg5	inhibits cell proliferation	(Liu et al. 2018)
circ-EIF6	Protein-coding	EIF6-224 aa/MYH9	Promotes proliferation and metastasis	(Li et al. 2022)
CircSEMA4B	Protein-coding	SEMA4B-211aa/PI3K/AKT	Negative regulator of PI3K/AKT signaling pathway	(Wang et al. 2022)
circEPSTI1	miRNA sponge	miR-4753/6809-BCL11A	Inhibits cell proliferation, migration, invasion and induces apoptosis	(Chen et al. 2018a, b)

## CircRNAs and the key hallmarks of breast cancer

Research has established that circRNAs play many diverse roles in BC progression. Some studies have discovered specific roles of circRNAs pertaining to certain hallmarks, such as angiogenesis, metastasis, or metabolic reprogramming (Yarmishyn et al. 2022). However, most circRNAs are involved in more than one hallmark of cancer (Table 2). Recent evidence on the roles of different circRNAs in BC hallmarks is summarized below.

### Role of circRNAs in metabolic reprogramming

Cancer cells exhibit heightened rates of cellular division and growth, which increases the cell’s metabolic demands and requirements. To satisfy these needs, cancer cells undergo metabolic reprogramming where they change the way they metabolize glucose, fatty acids, and amino acids (Cai et al. 2023). One prominent feature of metabolic reprogramming is the shift of the cancer cell from oxidative phosphorylation to glycolysis for the generation of energy, also known as the Warburg effect (Schiliro and Firestein 2021).

CircRNAs play crucial roles in metabolic reprogramming. For example, circSIPA1L3 boosts the rate of glycolysis in TNBC. This leads to an increase in the production of lactate and a subsequent attraction of tumor-associated macrophages and tumor metastasis (Liang et al. 2024a, b). Increased glutamine metabolism in BC has been extensively studied and characterized, offering targetable metabolic dependencies (Li et al. 2023) (Table 2). CircSEPT9 was found to upregulate solute carrier family 1 (neutral amino acid transporter), member 5 (SLC1A5) expression by sponging of miR-149-5p in TNBC (X. Wang et al. 2021a, b). Knockdown of circSEPT9 led to reduced glutamine uptake and cell proliferation and increased cell apoptosis (X. Wang et al. 2021a, b). Similarly, in TNBC, circ_0062558 was found to enhance glutamine metabolism by sponging of miR-876-3p which leads to enhanced expression of SLC1A5. This led to an increase of cell proliferation, survival, migration and invasion of TNBC cells (Yuan et al. 2022). For fatty acid metabolism, circMYC was found to be upregulated in TNBC, which binds to human antigen R (HuR) protein. This improves the binding affinity of HuR to the sterol regulatory element binding protein 1 (SREBP1) mRNA and increases its stability (S. Wang et al. 2023a, b). The enhanced stability of the SREBP1 mRNA leads to increased lipogenesis, ultimately leading to cancer progression (S. Wang et al. 2023a, b) (Table 2).

### Role of circRNAs in apoptosis

Normal cells activate apoptosis, or programmed cell death, once they accumulate increasing amounts of mutations or aberrations (Obeng 2021). However, one of the hallmarks of cancer is resistance to apoptosis, where the cells lose the ability to self-destruct (Neophytou et al. 2021). Studies have shown that circRNAs also play an influential role in enabling BC cells to escape apoptosis. CircZEB1, for instance, was found to be overexpressed in BC, increasing the sponging of miR-448, leading to a subsequent increase in eukaryotic elongation factor 2 kinase (eEF2k) (Pei et al. 2020). A recent study also discovered that circARHGER28 overexpression increases apoptosis of BC cells (Tao et al. 2024).

Another recent study found circSPECC1 to be overexpressed in BC tissues and that its downregulation led to an increase in apoptosis by increasing the expression of miR-1236-3p (Zhang et al. 2024). Conversely, the overexpression of circ_0058063 was found to enhance apoptosis by reducing miR-557 expression which leads to an increase in discs large-associated protein 5 (DLGAP5) expression (Zhu et al. 2024). DLGAP5 plays a role in polymerizing microtubules and formation of spindle fibers, which enables the cell to transition from the G2 phase of the cell cycle to the mitotic phase. In addition to the previous circRNAs, the overexpression of circCDYL was found to enhance apoptosis by binding to miR-190-3p and increasing the expression of tumor protein p53 inducible nuclear protein 1 (TP53INP1), a prominent tumor suppressor. Research has also revealed that inhibition of circSEPT9 increases apoptosis through the elevation of miR-625-5p expression (Table 2) (Shi et al. 2024).

### Role of circRNAs in the epithelial mesenchymal transition (EMT)

One of the initial stages of metastasis is the EMT, where the cancer cell changes its morphology and develops the capacity to separate from the extracellular matrix (ECM) and migrate into neighbouring tissues (Xu et al. 2024). Numerous studies have demonstrated that circRNAs play a role in the EMT process thus enabling or suppressing metastasis (Ashrafizadeh et al. 2024). One of the main mechanisms through which this takes place is by sequestering microRNAs and regulating the expression of oncogenes or tumor suppressor genes. A recent study highlighted the role of the circRNA for Jumonji C (JmjC) domain-containing (JMJD) demethylases (circ_JMJD1C) in EMT where it absorbs miR-182-5p leading to increased expression of JMJD1C and SRY-related HMG-box transcription factor 4 (SOX4) (Xu et al. 2024).

**Fig. 4:**
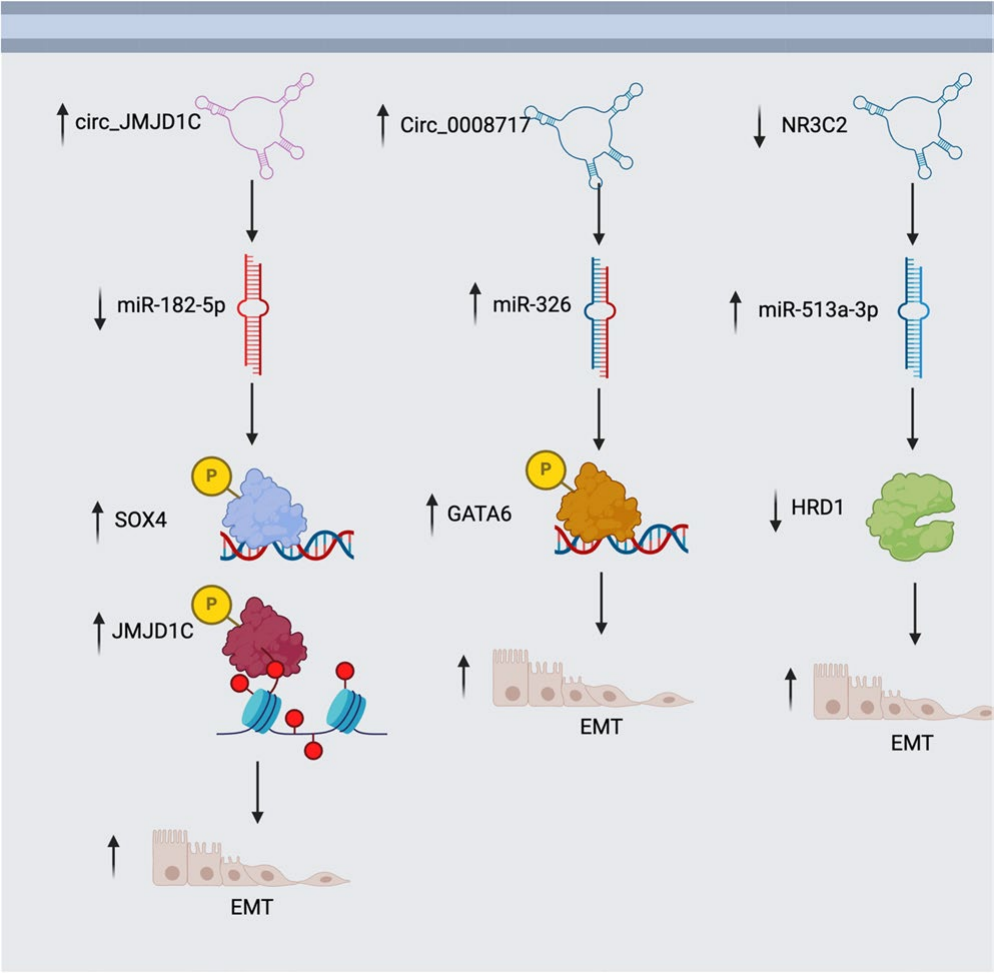
CircRNAs are implicated in epithelial mesenchymal transition (EMT) in breast cancer. CircJMJD1C upregulation leads to increased sponging of miR-182-5p, which increases the expression of SOX4 and JMJD1C, leading to increased EMT. Increased expression of circ_0008717 leads to increased expression of miR-326, negatively affecting the expression of GATA6, which, in turn, increases EMT. The expression of NR3C2 is downregulated in BC, leading to increased availability of miR-513a-3p. This reduces the expression of HRD1, and leads to increased EMT

While previous studies show that circ_JMJD1C can function as either a tumor promoter or suppressor in different cancers (Manni et al. 2022), SOX4 is a transcription factor that was shown to promote cellular migration by activating the transcription of chemokine Ligand 12 (CXCL12) (Tsai et al. 2020). Circ_JMJD1C has been demonstrated to not only regulate EMT, but that it also increases tumor growth and reduces programmed cell death (Xu et al. 2024). Circ_0008717 is another circRNA that plays a role in EMT as well as migration, angiogenesis, and invasion. Circ_0008717 achieves this effect by decreasing miR-326 leading to increase of GATA6. GATA6 is an oncogenic transcription factor that increases EMT by increasing the expression of Slug, another transcription factor (Fig. 4) (L. Yang and Chen 2023).

Another circRNA implicated in EMT is Nuclear Receptor Subfamily 3, Group C, Member 2 (NR3C2), which was found to be downregulated in TNBC. Its low expression in BC undermines the capacity of ERAD-associated E3 ubiquitin-protein ligase HRD1 (HRD1) to suppress tumor EMT by increasing the availability of miR-513a-3p (Fig. 4) (Fan et al. 2021). On the other hand, circANKS1B was found to be overexpressed in TNBC, which in turn leads to increased EMT by increasing the expression of transforming growth factor beta 1 (TGF-β1). TGF-β1 is a growth factor known to play a positive role in EMT by activating transcription regulators such as Smad2 and Smad3. In turn, Smad2 and Smad3 interact with transcription factors to activate the expression of mesenchymal genes and reduce the expression of epithelial genes (Xu et al. 2009). (Table 2).

### Role of circRNAs in breast cancer tissue invasion

Studies have found that circRNAs can also contribute to the capacity of BC cells to invade surrounding tissues. In TNBC, circ_0045881 was demonstrated to be downregulated. However, increasing the expression of circ_0045881 resulted in decreased expression of miR-214-3p, leading to decreased tumor-cell invasion capacity (Ren et al. 2024). Another study reported that there was lower circNFIB expression in BC tissues compared to control tissues, which was found to lead to increased metastasis. This observed increase in metastasis is thought to be mediated through reduced arachidonic acid (AA) synthesis (Zhong et al. 2024), which, in turn, leads to increased activation and expression of inflammatory cytokines and angiogenic factors. Tumor invasion of the lymph nodes was found to be correlated with increased expression of circ_0009910 and decreased expression of miR-145-5p (Abtin et al. 2024). Among the positive contributors is also circRREB1, which causes increased invasion, growth, and metastasis in BC by binding to guanine nucleotide-binding protein subunit beta-4 (GNB4) and activating the extracellular regulated kinase 1/2 (ERK1/2) pathway (Table 2) (H. Chen et al. 2024a, b).

Aside from the aforementioned circRNAs, increased circHSDL2 in BC patients was found to enhance the cancer’s capacity to invade in the matrigel and the transwell assays by sequestering miR-7978, leading to increased expression of Zinc Finger Protein 704 (ZNF704) and reduced expression of mammalian ste20-like kinase 2 (MST2) (Wang et al. 2024). MST2 is a well-known component of the Hippo pathway, which plays a role in cancer growth and invasion (Elemam et al. 2024; Wang et al. 2024b). Recent evidence also pointed to an increase in hsa_circ_0003528 expression in TNBC corresponding to a decrease in miR-215, leading to increased proliferative potential, migration abilities, and invasive capacities of TNBC cells (Table 2) (Chang et al. 2024).

Furthermore, circ_0104345 expression in BC cells was found to support their ability to not only survive apoptosis, but also to proliferate, move, and invade surrounding tissues. Circ_0104345 achieves this through sponging miR-876-3p, thereby increasing the expression of Zinc finger and BTB domain-containing protein 20 **(**ZBTB20), (Wu et al. 2023) (a transcription factor that reduces the expression of inhibitor of kappa B (IκB). This reduction in IκB leads to an increase of nuclear translocation of nuclear factor kappa B (NF-κB), which is thought to mediate an increase in matrix metalloprotease 2 and 9 (MMP2 and 9) and increased cellular migration (Stoyanov et al. 2023). In Human epidermal growth factor receptor 2 (HER2)-positive BC cells, upregulation of circEPSTI1 was found to promote cell proliferation, migration and invasion mediated by miR-145 sponging, leading to increased expression of Erb-B2 Receptor Tyrosine Kinase 3 (ERBB3), a tyrosine kinase receptor involved in the growth and survival of breast epithelial cells (Hamburger 2008; Zhang et al. 2022). In TNBC, circPRKC1 (hsa_circ_0067934) was demonstrated to upregulate WW domain binding protein **(**WBP2) and promote protein kinase B (AKT) phosphorylation through sponging of miR-545-3p leading to increased cell proliferation and migration (Table 2) (X. Wang, Song et al. 2022).

### Role of circRNAs in breast cancer metastasis

Regarding the involvement of circRNAs in metastasis, recent studies have shown that the downregulation of circFOXO, when observed in TNBC, is connected to metastasis of the breast tumor to lymph nodes resulting in a negative prognosis for patients. The downregulation of circFOXO resulted in an increase in the translocation of Wolf-Hirschhorn Syndrome Candidate 1 (WHSC1) to the nucleus, leading to an increase in the expression of zinc finger E-box binding homeobox 2 (Zeb2) and increased metastasis (Chen et al. 2024a, b). Studies suggest that Zeb2 increases metastasis by altering the expression of cell-cell junctions (Korpal et al. 2008). However, one study by Burks et al. (2021) suggests that Zeb2 does not alter the expression of epithelial or mesenchymal genes, although it does observe that Zeb2 plays a role in metastasis (Table 2).

One study demonstrated circIKBKB’s ability to promote the metastasis of BC to bone by activating the NF-κB pathway through the phosphorylation and inhibition of inhibitor of nuclear factor kappa B alpha (IκBα) (Xu et al. 2021). By activating the NF-κB pathway, circIKBKB promotes cancer-related inflammation and metastasis as a consequence (Liu et al. 2015). Other studies have demonstrated that circHIF1A contributes to the metastasis of BC to lymph nodes by binding to miR-149-5p and inhibiting cyclin-dependent kinase inhibitor 1 **(**P21), the main target of P53. The biogenesis of circHIF1A takes place through the activation of fused in sarcoma (FUS) protein, which is activated by nuclear factor 1 B (NFIB), which in turn increases the activity of the protein kinase B/signal transducer and activator of transcription 3 (AKT/STAT3) pathway. At the same time, circHIF1A itself modulates the activity of NFIB, creating a positive feedback loop that increases circHIF1A and increases the activity of AKT/STAT3 pathway (Table 2) (Chen et al. 2021; Zepeda-Enríquez et al. 2023).

Another recent study found that circ-0100519 expression was increased in BC, increasing metastasis by secretion into exosomes and an increase of the de-ubiquitination of nuclear factor-like 2 (NRF2) by the ubiquitin specific protease 7 (USP7) in macrophages (Zhuang et al., 2024). Moreover, circCAPG encodes a polypeptide which increases tumor growth by blocking the binding of the serine/threonine kinase (STK38) and SMAD-specific E3 ubiquitin protein Ligase 1 (SMURF1) in TNBC (Song et al. 2023). This results in reduced degradation of mitogen-activated protein kinase kinase kinase 2 (MEKK2) and a subsequent increased activation of the mitogen-activated protein kinase (MEKK2-MEK1/2-ERK1/2) pathway (Song et al. 2023). Studies indicate that this pathway can promote metastasis by upregulating matrix metalloproteases (Kciuk et al. 2022; Rocca et al. 2022). Moreover, circ_0059457 promotes metastasis as well as proliferation of BC cells through the sequestration of miR-140-3p, which increases the expression of ubiquitin-binding enzyme 2 C (UBE2C) (Huang et al. 2024). UBE2C was previously shown to promote tumor invasion and proliferation by activating protein kinase B/mammalian target of rapamycin (AKT/mTOR) pathway (Table 2) (Lu et al. 2021).

### Role of circRNAs in angiogenesis

As the tumor grows and migrates, it undergoes what is known as the “angiogenic switch”, where growth of blood vessels around the tumor is induced to provide the tumor with additional nutrients (Lugano et al. 2020). Recent studies have indicated that circRNAs also play a crucial role in angiogenesis. The upregulation of Hsa_circ_0001925 in TNBC tissues was found to play a role in angiogenesis, tumor growth and migration. This takes place through increasing the expression of Yin Yang 1 (YY1) by sequestering miR-1299 (Table 2) (Shen et al. 2023).

Previous studies stated that YY1 can contribute to angiogenesis and other hallmarks of cancer through the activation of epidermal growth factor receptor (EGFR) and human epidermal growth factor 2 (HER2) (Guo et al. 2020). Similarly, an increase in Hsa_circ_0008673 expression was associated with increased angiogenesis and tumor growth through elevated levels of GINS Complex Subunit 4 (GINS4) and reduced miR-578 absorption (Sun et al. 2023). Although the role of GINS4 in the angiogenesis of BC is unknown, it was previously shown to promote gastric cancer by activating Rac1 and cell division control protein 42 (CDC42) suggesting this might be its potential mechanism in increasing angiogenesis (Zhu et al. 2019). However, this requires further research (Table 2).

In addition to the aforementioned circRNAs, circRPPH1 was observed to increase angiogenesis of TNBC by sequestering miR-556-5p which enhances the expression of Yes-associated protein 1 (YAP1) and activation of Hippo signaling pathway (Zhou et al. 2020a, b). YAP1 is a key component of the Hippo pathway, which mediates the effects of angiogenic molecules such as vascular endothelial growth factor (VEGF) by regulating the transcription of key genes (Table 2) (Boopathy and Hong 2019).

In BC, circBRAF was demonstrated to be a key player in angiogenesis, metastasis, and proliferation of BC whereby it boosts the modification of histone H3K9me3 by increasing lysine demethylase 4B (KDM4B) recruitment (Lan et al. 2024). It also interacts with insulin-like growth factor 2 binding protein 3 (IGF2BP3). The histone modification in turn leads to increased expression of matrix metalloprotease 9 (MMP9) and ADAM metallopeptidase with thrombospondin type 1 motif 14 (ADAMTS14), enzymatic components of the ECM (Table 2) (Lan et al. 2024).

In another study, circZEB1 was found to be upregulated in BC cells and that its downregulation reduced angiogenesis and invasion potential of the cancer cells by increasing miR-337-3p and subsequently inhibiting the expression of O-GlcNAc transferase (OGT). This inhibits the O-GlcNAcylation of YBX1 leading to its degradation (D. Wang, Chen et al. 2024a, b). Although the exact mechanism by which YBX1 affects BC is not known, several oncogenes were found to be targets of YBX1, such as cellular myelocytomatosis oncogene (c-myc) and AKT, among others. More research is needed to identify how YBX1 can promote angiogenesis through the transcriptional regulation of oncogenes. Another circRNA that was found to play a positive role in angiogenesis is Circ_ 0002496 (Table 2) (Yang et al. 2023).

**Table 2:** CircRNAs in breast cancer and their targets

CircRNA	Regulation	Target miRNA	Pathway	Reference
Circ_JMJD1C	Up	miR-182-5p	JMJD1C and SOX4	(Xu et al. 2024)
Circ_0008717	Up	miR-326	GATA6	(L. Yang and Chen 2023)
NR3C2	Down	miR-513a-3p	HRD1	(Fan et al. 2021)
CircFOXO	Down	WHSC1	Zeb2	(Chen et al. 2024a, b)
CircIKBKB	Up	IκBα	NF- κB pathway	(Xu et al. 2021)
CircHIF1A	Up	miR-149-5p	AKT/STAT3 pathway	(Chen et al. 2021; Zepeda-Enríquez et al. 2023)
Circ-0100519	Up	USP7	USP7 and NRF2	(Zhuang et al., 2024)
CircCAPG	Up	STK38 SMURF1	MEKK2-MEK1/2-ERK1/2 pathway	(Song et al. 2023)
Circ_0059457	Up	miR-140-3p	UBE2C	(Huang et al. 2024)
Hsa_circ_0001925	Up	miR-1299	YY1	(Shen et al. 2023)
Hsa_circ_0008673	Up	miR-578	GINS4	(Sun et al. 2023)
CircRPPH1	Up	miR-556-5p	YAP1/Hippo signaling pathway	(Y. Zhou et al. 2020a, b)
CircBRAF	Up	KDM4B IGF2BP3	MMP9 ADAMTS14	(Lan et al. 2024)
CircZEB1	Up	miR-337-3p	YBX1 and OGT	(D. Wang, Chen et al. 2024a, b)
Circ_ 0002496	Up	miR-433-3p	YWHAZ Bax And Bcl-2	(Yang et al. 2023)
Circ_0045881	Down	miR-214-3p	Unknown	(Ren et al. 2024)
CircNFIB	Down	Encodes a 56 amino acid protein	Arachidonic acid synthesis	(Zhong et al. 2024)
Circ_0009910	Up	miR-145-5p	MUC1	(Abtin et al. 2024)
CircRREB1	Up	GNB4	ERK1/2 pathway	(H. Chen et al. 2024a, b)
CircHSDL2	Up	miR-7978	ZNF704 and MST2	(Wang et al. 2024)
hsa_circ_0003528	Up	miR-215	Unknown	(Chang et al. 2024)
Circ_0104345	Up	miR-876-3p	ZBTB20	(Wu et al. 2023)
CircZEB1	Up	miR-448	eEF2k	(Pei et al. 2020)
CircARHGER28	Up	Unknown	PI3K/AKT/mTOR pathway	(Tao et al. 2024)
CircSPECC1	Up	miR-1236-3p	CBX8	(Zhang et al. 2024)
CircCDYL	Down	miR-190a-3p	TP53INP1	(Wang et al. 2020)
Circ_0058063	Up	miR-557	DLGAP5	(Zhu et al. 2024)
CircSEPT9	Up	miR-625-5p	PTBP3	(Shi et al. 2024)
CircSIPA1L3	Up	miR-665 IGF2BP3	SLC16A1 RAB11A (glycolysis)	(Yuan et al. 2022)
CircPRKC1	Up	miR-545-3p	AKT phosphorylation	(X. Wang, Song et al. 2022)

## CircRNAs as potential diagnostic markers in breast cancer

**Fig. 5:**
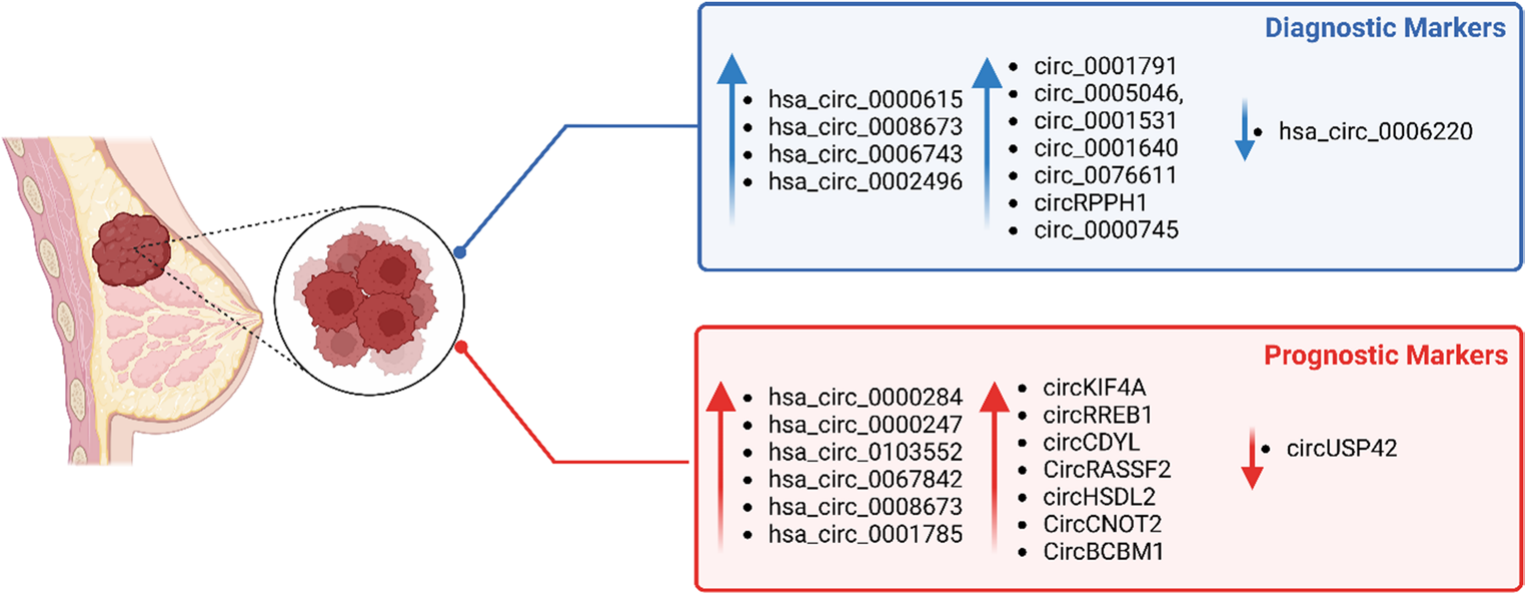
CircRNAs as Potential Prognostic and Diagnostic Markers in Breast Cancer. The blue box lists diagnostic markers, including upregulated (e.g., hsa_circ_0000615, circ_0005046) and downregulated (e.g., hsa_circ_0006220) circRNAs. The red box lists prognostic markers, including upregulated (e.g., hsa_circ_0000284, circCDYL) and downregulated (e.g., circUSP42) circRNAs. Arrows indicate expression changes. Data reflects reported expression patterns in breast cancer tissue

CircRNAs have been identified as promising biomarkers and can be used to diagnose BC. They are found in abundance and are expressed in various tissues. CircRNAs can be obtained from body fluids (blood, plasma, and exosomes) and are more stable than linear RNA as they are more resistant to exonuclease-mediated degradation due to their covalent closed continuous loop structure (Fontemaggi et al. 2021). Studies have shown that the dysregulation of circRNAs can be used as tools for early detection of BC. Numerous studies have demonstrated the dysregulation of circRNAs in BC tissues and their potential utility as diagnostic and prognostic markers. For example, hsa_circ_0000615 was found to be highly expressed in blood samples and the receiver operator characteristic (ROC) showed the value of the area under curve (AUC) was 0.904, with a sensitivity of 76.8%, and specificity of 88.4% (Liu et al. 2021a, b, c). The authors conducted a dual comparison approach that utilized blood samples drawn from age-matched healthy control volunteers as well as adjacent normal tissues from the same BC patients. The study illustrated that hsa_circ_0000615 had superior sensitivity and specificity in comparison to traditional tumor markers (e.g., including CA153, CA125, and CEA), highlighting its potential as a more accurate biomarker in diagnosing BC (Liu et al. 2021a, b, c). hsa_circ_0008673 was found to be upregulated in plasma samples of BC patients (Y. Hu et al. 2020a, b). Conversely, hsa_circ_0006220 was significantly down-regulated, a 27-fold decrease compared to healthy tissues, in BC tissues (C. Liu et al. 2021a, b, c). Furthermore, three circRNAs: circ_0000745, circ_0001531, circ_0001640 were identified to be strongly upregulated in whole blood samples of BC patients, suggesting their potential use as novel biomarkers (Table 3) (Fig. 5) (Wang et al. 2021a, b).

Moreover, circ_0005046 and circ_0001791 were found to be upregulated in tissues of BC patients, illustrating their potential as biomarkers for early cancer detection (Ameli-Mojarad et al. 2021). It is also worth discussing that hsa_circ_0006743 and hsa_circ_0002496 were reported to be upregulated in BC cells obtained from tissues, supporting their promising potential as biomarkers for early detection of BC (Rao et al. 2021). Additionally, circ_0076611, was detected in serum samples since it is released from TNBC cells into exosomes (Turco et al. 2022). Overall, the differential expressions of circRNAs demonstrate promising potential to be utilized as biomarkers for early detection of BC (Table 3) (Fig. 5).

Recently the roles of circ_0001522, circ_0001278, and circ_0001801 as promising diagnostic and prognostic biomarkers were investigated by Awata et al. (2025) and were found to be positively correlated with poor-relapse free survival. Moreover, the circRNAs demonstrated significant overexpression in tumor tissues in comparison to the adjacent sample tissues of the same patient, indicating the critical role of these circRNAs as key breast cancer hallmarks in promoting TNBC proliferation, migration and 3D-growth and suggesting their role as a potential biomarker (Table 3).

**Table 3:** CircRNAs as diagnostic biomarkers in breast cancer

CircRNA	Expression	Source	Reference
CircRPPH1	Upregulated	Tissue	(Huang et al. 2021a, b)
hsa_circ_0000615	Upregulated	Blood	(Liu et al. 2021a, b, c)
hsa_circ_0008673	Upregulated	Plasma	(Y. Hu et al. 2020a, b)
hsa_circ_0006220	Downregulated	Tissue	(Liu et al. 2021a, b, c)
circ_0000745 circ_0001531 circ_0001640	Upregulated	Blood	(Wang et al. 2021a, b)
circ_0005046 circ_0001791	Upregulated	Tissue	(Ameli-Mojarad et al. 2021)
hsa_circ_0006743 hsa_circ_0002496	Upregulated	Tissue	(Rao et al. 2021)
circ_0076611	Upregulated	Serum	(Turco et al. 2022)
circ_0001522 circ_0001278 circ_0001801	Upregulated	Serum	(Awata et al. 2025)

## CircRNAs as potential prognostic markers in breast cancer

The upregulation of various circRNAs, as discussed thus far, suggests their potential prognostic value in BC. For instance, circKIF4A was found to be significantly upregulated in TNBC, which correlated to large tumor size and lymph node metastasis (Tang et al. 2019). Knockdown of this circRNA also led to a significant decrease of TNBC cell proliferation, suggesting its role in cancer progression.

In another study, circRREB1, a novel circRNA that is upregulated in BC, was identified to promote increased tumor growth and metastasis through extracellular signal-regulated kinase 1/2 (Erk 1/2) signaling pathway by interaction with GNB4 and is associated with poor prognosis in BC patients (H. Chen et al. 2024a, b). The elevated expression of circRREB1 correlates with advanced stages of BC and shorter survival times according to the Kaplan-Meier survival analysis. On the other hand, the expression of circUSP42 was strongly downregulated in TNBC tissues (Yu et al. 2020). The downregulation was associated with lymph node metastasis, suggesting that the dysregulation of circUSP42 is involved in the progression of TNBC (Table 4) (Fig. 5) (Yu et al. 2020).

Hsa_circ_0000284 was found to be upregulated in tissues of BC patients, which also promotes cell proliferation, migration, and invasion of BC by regulating signaling pathways that are crucial for cancer progression. Upregulation of this circRNA predicted a poor prognosis in BC patients (Luo et al. 2021). Similarly, another circRNA, circHMCU (hsa_circ_0000247) also demonstrated an upregulation in BC tissues with high metastatic potential. This circRNA enhances BC cell growth, increases migration and invasion, and suppresses the expression of let-7 MicroRNA. Downregulation of let-7 can lead to increased expression of oncogenes such as *MYC*, *HMGA2*, and *CCND1* (Song et al. 2020).

In another study, hsa_circ_0103552 was found to be upregulated in BC cells and associated with cancer cell proliferation and cell metastasis resulting in poor prognosis for BC patients (Huang et al. 2021a, b). Furthermore, hsa_ circ_0067842 was also highly upregulated in BC tissues (Table 4) (Fig. 5) (Li et al. 2023). This circRNA is strongly associated with poor prognosis, as it can promote cancer cell proliferation and metastasis as well as enhance the migration and invasion of BC cells, thereby contributing to immune escape.

Likewise, hsa_circ_0008673 located in the plasma of BC patients was found to be upregulated compared to normal cells. This circRNA was also reported to be associated with increased tumor size and distant metastasis (Y. Hu et al. 2020a, b). Knockdown of hsa_circ_0008673 led to the disruption of tumor cell proliferation and metastasis. The study suggests that hsa_circ_0008673 could be used as a biomarker and that its presence contributes to poor prognosis in patients. In BC tissues, circCDYL was found to be dysregulated in comparison to Healthy tissues and was found to be elevated by 3.2 folds. The increased expression of this circRNA has been associated with increased cell autophagy and BC malignancy. Furthermore, the increased levels of circCDYL are correlated with poor prognosis in BC patients (Liang et al. 2020).

CircRASSF2 was found to be significantly elevated in both serum and tissues of BC patients. Increased levels of circRASSF2 have also been associated with enhanced tumor size, distant metastasis and lymph node metastasis, which lead to a decrease in overall survival for BC patients. Targeting circRASSF2 will allow for novel therapeutic approach and novel prognostic biomarkers (Table 4) (Fig. 5) (Zhong et al. 2021).

Additionally, (Liang et al. 2024a, b) reported that CircGSK3β plays a pivotal role in promoting BC progression and aided in cell immune progression, highlighting its potential as a promising prognostic biomarker. Additionally, the study found that it was highly expressed in various BC cell lines including MDA-MB-231 and MCF7 compared to normal control cell lines. Knockdown of the circRNA led to a substantial reduction in aggressive BC progression and migration capabilities. Moreover, its role in immune evasion was highlighted by the upregulation of PD-L1 through miR-338-3p/PRMT5 interaction. PD-L1 is expressed upon the binding of PRMT5, a type II enzyme, to its promoter increasing its transcription through histone H3K4 trimethylation (H3K4me3) which leads to cancer invasion and immune evasion (Table 4) (Liang et al. 2024a, b).

Recent findings have identified circ_0022382 as an oncogenic circRNA that is significantly upregulated in BC tissues and cell lines (MDA-MB-231, MCF-7) compared to adjacent normal tissues. Knockdown of this circRNA resulted in the inhibition of the proliferation and migration of BC cells both in vitro and in vivo (Liu et al. 2024). Circ_0022382 serves as a promising biomarker in BC diagnosis due to its essential role in cancer cell progression. Furthermore, the study also revealed that circ_0022382 can act as a sponge for let-7a-5p miRNA which is often downregulated in BC cells and promotes downstream oncogenic signaling. The circRNA affects the PI3K/AKT/mTOR signaling pathway as well as the upregulation of SLC7A11, essential for tumor metabolism. Upon knocking down this circRNA, reduced phosphorylation of AKT and SLC7A11 was observed. Contribution to disulfidptosis was also observed, a type of cell apoptosis that is triggered by the accumulation of cystine and altered glucose metabolism. The study also noted that the expression of circ_0022382 is promoted upstream by EIF4A3, an RNA helicase that is often reported to be expressed in BC cells. Targeting pathways such as PI3K/AKT/mTOR and SLC7A11 could potentially serve as a novel therapeutic approach in BC treatment (Table 4) (Liu et al. 2024).

Moreover, Yi et al. (2025) recently characterized circFOXK2 as a hallmark driver in advancing the proliferation, and aggression of ER-positive BC. The increase of circFOXK2 expression in both cell lines and BC tissues is associated with CCND1, which is a key regulator in cell cycle transition. Unlike most circRNAs mentioned in this review, circFOXK2 does not act as a sponge for miRNA, rather, it directly binds to the CCND1 mRNA, specifically, the 3’ UTR region through RNA-RNA pairing. The circRNA also recruits ELAVL1 which is an RNA-binding protein that stabilizes CCND1 mRNA as well as increasing its expression. Elevation of CCND1 protein levels can activate the CCND1–CDK4/6–pRB–E2F signaling cascade which in turn promotes the transcription of *E2F* target genes that promote tumor growth. The study also suggested that circFOXK2 can serve as a potential therapeutic target using antisense oligonucleotide (ASO-circFOXK2) which can lead to the suppression of ER-positive BC growth and can be used synergistically alongside tamoxifen. The study discussed that the combination of ASO-circFOXK2 and tamoxifen was capable of resensitizing tamoxifen resistant ER-positive BC cells (Table 4).

**Table 4:** CircRNAs as prognostic biomarkers in breast cancer

CircRNA	Expression	Source	Prognosis	Reference
circKIF4A	Upregulated	Tissue	Poor	(Tang et al. 2019)
circRREB1	Upregulated	Tissue	Poor	(H. Chen et al. 2024a, b)
circUSP42	Downregulated	Tissue	Poor	(Yu et al. 2020)
hsa_circ_0000284	Upregulated	Tissue	Poor	(Luo et al. 2021)
hsa_circ_0000247	Upregulated	Tissue	Poor	(Song et al. 2020)
hsa_circ_0103552	Upregulated	Tissue	Poor	(Huang et al. 2021a, b)
hsa_circ_0067842	Upregulated	Tissue	Poor	(Li et al. 2023)
hsa_circ_0008673	Upregulated	Plasma	Poor	(Y. Hu et al. 2020a, b)
circCDYL	Upregulated	Tissue	Poor	(Liang et al. 2020)
circRASSF2	Upregulated	Serum	Poor	(Zhong et al. 2021)
circHSDL2	Upregulated	Serum	Poor	(S. Yang and Tang 2020)
hsa_circ_0001785	Upregulated	Plasma	Poor	(Yin et al. 2018)
circCNOT2	Upregulated	Plasma	Poor	(Smid et al. 2019)
circBCBM1	Upregulated	Serum	Poor	(Fu et al. 2021)
circGSK3β	Upregulated	Tissue	Poor	(Liang et al. 2024a, b)
circ_0022382	Upregulated	Tissue	Poor	(Liu et al. 2024)
circFOXK2	Upregulated	Tissue	Poor	(Yi et al. 2025)

## The role of circRNAs in breast cancer cells’ drug resistance

Drug resistance is a major cause of cancer therapy failure; however, the underlying mechanisms remain to be fully elucidated. CircRNAs can either promote or inhibit resistance to traditional chemotherapy, endocrine therapy, and even targeted therapies across various types of tumors, suggesting their potential as a novel focus for research into overcoming drug resistance. This highlights the promise of circRNAs as potential therapeutic targets to combat chemoresistance in BC (Misir et al. 2022). The following sections highlight the involvement of circRNAs in resistance to commonly used BC drugs in three major categories: chemotherapy, endocrine therapy, and targeted therapy.

### Chemotherapy resistance

Chemotherapy is vital for breast cancer treatment, but chemoresistance limits its efficacy. CircRNAs regulate resistance by modulating apoptosis, autophagy, and drug efflux, often acting as ceRNAs to sequester miRNAs, thereby influencing drug resistance-associated gene expression (Ghazimoradi and Babashah 2022; He et al. 2021a, b).

#### Adriamycin (Doxorubicin)

Doxorubicin, also known as adriamycin (ADM), belongs to a class of chemotherapy drugs known as anthracyclines. It works by slowing or halting the growth of cancer cells through the inhibition of topoisomerase 2 (Granados-Principal et al. 2010). This medication is commonly used in the treatment of Estrogen receptor–positive breast cancer (ER + BC) (Pritchard et al. 2012). ADM is an antibiotic that does not target a specific phase of the cell cycle. ADM functions by blocking the synthesis of DNA and RNA, thereby impeding tumor progression (He et al. 2021a, b).

##### Hsa_circ_0006528

Researchers have identified that hsa_circ_0006528 is elevated in (ADM)-resistant BC cells (Afzal et al. 2022). Previous studies indicate that hsa_circ_0006528 sponges miR-7-5p, leading to Raf1 activation, leading to the activation of the downstream MAPK/ERK signaling pathway, making it a potential therapeutic target for reducing recurrence and metastasis (He et al. 2021a, b). Additionally, it is also considered a promoter of the RhoA/ROCK pathway by sponging miR-1236-3p, increasing CHD4 expression (Table 5) (Ghazimoradi and Babashah 2022).

##### Hsa_circ_0001839 (CircKDM4C)

**Fig. 6:**
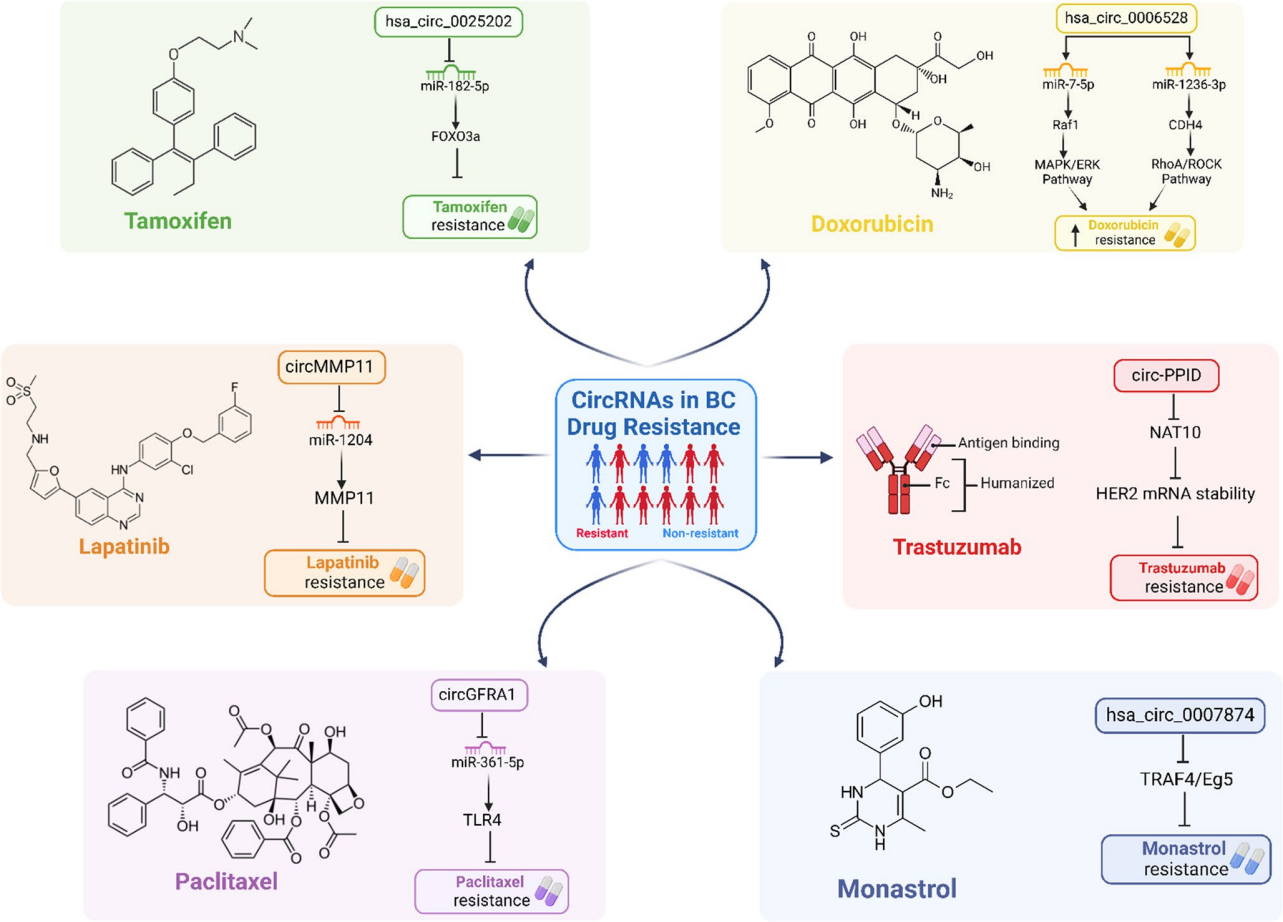
The Role of CircRNAs in Breast Cancer Cells’ Drug Resistance. This figure illustrates the involvement of circRNAs in mediating resistance to common breast cancer treatments, including doxorubicin, tamoxifen, paclitaxel, monastrol, lapatinib, and trastuzumab

ADM-resistant cells were found to have lower levels of circKDM4C compared to their sensitive parental counterparts (De Palma et al. 2022). The knockdown of circKDM4C led to a significant increase in ADM resistance, while its overexpression resulted in decreased resistance by interacting with miR-548p. This interaction results in upregulation of phenazine biosynthesis-like domain-containing (PBLD) expression, which was established to function as tumor suppressor in BC tumorigenesis. Additionally, the overexpression of circKDM4C reduced the migration and invasion capabilities of ADM-resistant BC cells, highlighting the connection between metastasis and chemoresistance. (Fig. 6) (Table 5) (Liang et al. 2021a, b).

##### Circ_0085495

Circ_0085495, located in the cytoplasm, is overexpressed in ADM-resistant BC. Silencing it has been shown to reduce ADM resistance by modulating the miR-873-5p/integrin β1 signaling pathway (Xie and Zheng 2022). miR-873-5p inhibits integrin β1, a protein associated with cancer cell metastasis and resistance. Therefore, lowering circ_0085495 levels can help reduce both drug resistance and cancer cell growth (Table 5) (Ghazimoradi and Babashah 2022).

#### Paclitaxel (PTX)

Paclitaxel (PTX) was first segregated from the bark of pacific yaw trees; it was identified as a tetracyclic diterpenoid compound. As it is known for its low toxicity, high effectiveness, and wide-ranging anti-cancer properties, PTX has been widely used against BC. The mechanism of action of PTX involves various pathways in which it affects cellular processes resulting in apoptosis (Y.-H. Yang et al. 2020).

##### CircGFRA1

CircGFRA1 has been identified as a key factor contributing to the resistance to PTX in TNBC cells by sponging miR-361-5p, which normally inhibits TLR4, a protein associated with cancer cell survival and drug resistance. When circGFRA1 is knocked down, the resistance of TNBC cells to PTX is reduced due to a decrease in TLR4 expression (Lyu et al. 2021). Thus, circGFRA1 can function as a regulator of miR-361-5p/TLR4 and by blocking TLR4, the responsiveness of TNBC cells to PTX could be significantly enhanced, improving their sensitivity to the drug in BC treatment (Fig. 6) (Table 5) (Misir et al. 2022).

##### CircBACH1

CircBACH1 has been shown to promote colorectal cancer progression by lowering let-7a-5p levels, leading to elevated expression of CREB5, a transcription factor involved in eukaryotic gene regulation. CREB5, a protein-coding gene, plays a key role in controlling cellular processes through its function as a transcription factor (J. Li et al. 2020a, b). However, the way exosomal circBACH1 influences chemoresistance and metastasis in BC are still unclear. Recent research revealed that circBACH1 levels are higher in exosomes from PTX-treated BC cells and BC tissues. The ability of circBACH1 to sponge miR-217, leading to the upregulation of G3BP2 expression, highlights a potential therapeutic target for combating PTX resistance and BC progression through the circBACH1/miR-217/G3BP2 axis. Moreover, studies confirmed that reducing circBACH1 levels enhances sensitivity to PTX by inhibiting the viability, stemness, migration, and angiogenesis of BC cells, making it a potential multifaceted target in BC management (Table 5) (He et al. 2024; J. Li et al. 2020a, b).

#### Monastrol

Monastrol causes mitotic arrest at the G2/M phase by targeting the kinesin-5 motor protein, known as Eg5 (KIF11). By inhibiting its basal and microtubule functions, monastrol disrupts Eg5’s ability to maintain bipolar spindles, leading to cell apoptosis (Galeano et al. 2024). However, its drug resistance limits the application in BC therapy (He et al. 2021a, b).

Hsa_circ_0007874 (Circ_MTO1).

Circ_MTO1 was found to be downregulated in cell lines that have developed resistance to monastrol. It influences the TRAF4/Eg5 axis by binding TRAF4 to the Eg5 gene, which in turn inhibits BC cell activity and enhances the cytotoxic effects of monastrol (He et al. 2021a, b). When circMTO1 is silenced, cancer cell viability increases. Conversely, overexpression of circMTO1 leads to increased cell death by sequestering TRAF4 in the cytoplasm during monastrol treatment, thereby preventing Eg5 expression, which is essential for bipolar spindle separation (Das et al. 2021). Therefore, circMTO1 may play a crucial regulatory role in BC, and regulating its levels could be a promising approach to overcoming chemoresistance (Fig. 6) (Table 5) (W. Liu et al. 2021a, b, c).

### Endocrine therapy resistance

Endocrine therapy is essential for HR-positive patients and is a widely used and effective treatment. However, resistance to this therapy still occurs in about 20–30% of patients (Cen et al. 2023). Recent studies suggest that circRNAs are closely linked to endocrine therapy response, influencing resistance by modulating estrogen receptor signaling and apoptosis pathways (Y. Liang et al., 2019; Yao et al. 2020).

#### Tamoxifen

Tamoxifen (TAM) is a crucial treatment for women with estrogen receptor-positive (ER+) BC. It is both affordable and lifesaving, with minimal side effects for most individuals (Shagufta and Ahmad 2018). However, drug resistance limits its efficacy, making it essential to find ways to overcome tamoxifen resistance in BC (Yao et al. 2020).

##### CircBMPR2

CircBMPR2 expression is markedly reduced in tamoxifen-resistant BC cells and tissues, indicating a potential tumor-suppressive function in this setting. Functional studies have demonstrated that overexpression of circBMPR2 not only suppresses BC cell motility but also enhances their sensitivity to tamoxifen by promoting apoptosis, thereby improving therapeutic efficacy (Ghazimoradi and Babashah 2022). In contrast, circBMPR2 knockdown promotes tamoxifen resistance through the inhibition of tamoxifen-induced apoptosis (Liang et al. 2019b) Additionally, CircBMPR2 diminishes the motility of BC cells, hence constraining their metastatic capability, and enhances their sensitivity to tamoxifen by facilitating tamoxifen-induced apoptosis. The silencing of circBMPR2 enhances tamoxifen resistance by suppressing apoptosis. miR-553 enhances proliferation, metastasis, and tamoxifen resistance while establishing a negative feedback loop with circBMPR2, somewhat mitigating its effects (Table 5) (Y. Liang et al., 2019).

##### Hsa_circ_0025202

Hsa_circ_0025202 was found to be downregulated in BC tissues, particularly in tamoxifen (TAM)-resistant BC cells. Loss-of-function experiments revealed that when hsa_circ_0025202 is silenced in BC cells, the IC_50_ value for TAM increases, indicating enhanced resistance to the drug (Li et al. 2021). Hsa_circ_0025202 can act as a miRNA sponge, specifically targeting miR-182-5p. The interaction between miR-182-5p and FOXO3a plays a key role in cancer development and progression by influencing pathways that regulate the cell cycle and enhance cell survival (He et al. 2021a, b). FOXO3a, a member of the forkhead box O (FOXO) transcription factors, is known as a tumor suppressor in various cancers. It was shown to enhance TAM sensitivity by functioning as a competing endogenous RNA (ceRNA) for miR-182-5p, which modulates the expression and activity of FOXO3a, thereby potentially inhibiting tumor progression in hormone receptor-positive BC patients undergoing TAM therapy (Fig. 6) (Table 5) (Sang et al. 2019).

##### Circ_UBE2D2

Circ_UBE2D2 is elevated in TAM-resistant BC tissues. Exosomes from these resistant cells are rich in circ_UBE2D2 and can transfer this RNA to BC cells, increasing their TAM resistance (Hu et al. 2020a, b). However, overexpression of miR-200a-3p inhibits circ_UBE2D2, leading to lower vimentin levels and increased E-CAD and ERα expression, which are associated with reduced drug resistance (Ghazimoradi and Babashah 2022). Therefore, reducing circ_UBE2D2 may offer a potential strategy to overcome hormone therapy resistance (Table 5).

### Targeted therapy resistance

Targeted therapy is a cancer treatment approach that focuses on specific genes or proteins driving tumor growth. Unlike traditional chemotherapy, it selectively attacks cancer cells while sparing normal cells. In BC treatment, targeted therapies such as HER2 inhibitors and tyrosine kinase inhibitors (TKIs) play a paramount role. These therapies align with personalized medicine by tailoring treatment to a patient’s molecular profile, improving precision and efficacy. However, circRNAs contribute to resistance by interacting with oncogenic pathways, underscoring the need to develop strategies that directly target circRNAs to overcome resistance and enhance the effectiveness of personalized therapies (Wu et al. 2021).

#### Lapatinib

Lapatinib is a small molecule dual tyrosine kinase inhibitor (TKI) that targets Human Epidermal growth factor Receptor 1 (HER1) and Human Epidermal growth factor Receptor 2 (HER2). It works by binding reversibly to the cytoplasmic ATP-binding site of the tyrosine kinase domains of HER1 and HER2, thereby blocking the activation of downstream cascades (Voigtlaender et al. 2018).

##### Circ-MMP11

In human BC tissue, circMMP11 is significantly elevated and associated with lymph node metastasis and clinical stage. The circMMP11/miR-1204/MMP11 axis promotes BC progression through the ceRNA mechanism (Li et al. 2020a, b). Additionally, exosomal circMMP11 levels are higher in lapatinib-resistant (LR) BC tissues. Knockdown of circMMP11 enhances lapatinib sensitivity and induces apoptosis in resistant BC cells. Notably, circMMP11 is transferable via exosomes, highlighting its role in cancer progression. Its contribution to lapatinib resistance, cell growth, and metastasis is potentially mediated by the miR-153-3p/ANLN axis in LR BC cells (Fig. 6) (Table 5) (Wu et al. 2021).

#### Trastuzumab

Trastuzumab is a monoclonal antibody used primarily to treat BC and certain types of gastric cancer. It specifically targets the HER2 protein (Maadi et al. 2021).

##### Circ-PPID

Low expression levels of circ- peptidylprolyl isomerase D (circ-PPID) have been identified to contribute to trastuzumab resistance in HER2 BC. The restoration of circ-PPID was demonstrated to significantly enhance trastuzumab sensitivity by promoting HER2 mRNA decay, mediated by circ-PPID binding to N-acetyltransferase 10 (NAT10) in the nucleus and inhibition of the interaction between NAT10 and HER2 mRNA, reducing N4-acetylcytidine (ac4C) modification on HER2 exon 25 (Wang et al. 2024a, b, c). These findings highlight the potential therapeutic role of circRNAs in overcoming cancer therapy resistance. Therefore, a comprehensive investigation of how miRNAs and circRNAs regulate responses to targeted therapies could offer crucial insights for enhancing BC-targeted treatments (Fig. 6) (Table 5).

**Table 5:** CircRNAs involved in drug resistance mechanisms in breast cancer

CircRNA	miRNA sponges	Target Pathway	Dysregulation	Drug resistance	Mechanism	References
hsa_circ_0006528	miR-7-5p	raf1 & MAPK/ERK	Upregulation	ADM	Increases resistance of BC to ADM by interacting with mir-7-5p and raf1	(Gao et al. 2019)
hsa_circ_0006528	miR-1236-3p	RhoA/ROCK	Upregulation	ADM	Increases CH4 expression	(Ghazimoradi and Babashah 2022)
hsa_circ_0001839	miR-548p	PBLD	Downregulation	ADM	Increases resistance of BC to ADM by sponging mir-548p to increase the PBLD expression	(De Palma et al. 2022)
Circ_0085495	miR-873-5p	Integrin β1	Upregulation	ADM	Promotes resistance by sponging miR-873-5 which inhibits integrin β1	(Ghazimoradi and Babashah 2022; Xie and Zheng 2022)
Circ_0001667	miR-4458	NCOA3	Upregulation	ADM	Promotes resistance by binding to miR-4458, which typically suppresses NCOA3. NCOA3 is a protein that helps cancer cells grow and resist endocrine therapies	(Cui et al. 2022; Ghazimoradi and Babashah 2022)
Circ_0001667	miR-193a-5p	Rap2A	Upregulation	ADM	Leads to the downregulation of Rap2A mRNA and protein levels by targeting the 3’UTR of Rap2A	(Xu et al. 2023)
CircRNA-CREIT		PKR/eIF2α	Downregulation	ADM	CircRNA-CREIT enhances HACE1-mediated PKR degradation, inhibiting the PKR/eIF2α signaling axis and SG formation	(Wang et al. 2022)
hsa_circ_0025202	miR-182-5p	FOXO3a	Downregulation	TAM	Exert tumor inhibition and TAM sensitization effects via the miR-182-5p/FOXO3a axis	(He et al. 2021a, b)
Circ_UBE2D2	MiR-200a-3p	ERα	Upregulation	TAM	Resist TAM by sponging miR-200a-3p, miR-200a-3p	(He et al. 2021a, b)
CircBMPR2	MiR-553	USP4	Downregulation	TAM	Reduces the inhibition of apoptosis induced by tamoxifen and regulate miR-553	(Y. Liang et al., 2019)
circRNA-SFMBT2		ERα	Upregulation	TAM	CircRNA-SFMBT2 recruit RNF181 to ERα-positive cells, forming a complex that prevents ERα from being degraded through ubiquitination	(Z. Li et al. 2023a, b, c, d)
circBACH1	miR-217	G3BP2	Upregulation	PTX	PTX-induced exosomal circBACH1 promoted stemness and migration of BC cells by sponging miR-217 to upregulate the expression of G3BP2	(He et al. 2024)
CircGFRA1	miR-361-5p	TLR4	Upregulation	PTX	CircGFRA1 sponges miR-361-5p, preventing it from inhibiting TLR4, a protein linked to cancer cell survival and drug resistance	(Misir et al. 2022)
circ-MMP11	miR-153-3p	ANLN	Upregulation	Lapatinib	Elevates lapatinib resistance by regulating the miR-153-3p/ANLN axis in breast cancer cells	(Wu et al. 2021)
hsa_circ_0007874		TRAF4/Eg5	Downregulation	Monastrol	Sequesters TRAF4 from binding to EG5 protein	(W. Liu et al. 2021a, b, c).

## Computational approaches for circRNAs detection and validation

Detecting circRNA from RNA sequencing data primarily involves two key approaches: candidate-based and segmented read-based methods. The candidate-based method relies on a predefined list of potential back-splicing junction (BSJ) sequences, which is usually created by combining all pairs of known annotated exons within a gene. However, this approach is limited to species with annotated genomes and can only identify circRNAs that share the same splicing sites as linear RNAs. On the other hand, the segmented read-based method involves breaking down unmapped sequencing reads into shorter fragments, which are then realigned to the reference genome (Rebolledo et al. 2023).

When predicting the sub-cellular localization of circRNAs, methods utilizing residue frequency-based sequence descriptors and tree-based classifiers have proven more effective.(Asim et al. 2022). Among the various tools available, Circall stands out for its high sensitivity and precision in simulation studies, surpassing other circRNA detection methods in experimental dataset analysis. The efficiency of Circall is further enhanced by its use of an ultra-fast quasi-mapping algorithm, making it particularly well-suited for analysing large datasets (Nguyen et al. 2021).

LLCDC which can preserve local information During the encoding process was used in predicting circRNAs associated with BC with an AUC value of 0.9177 (Ge et al. 2020). Furthermore, SIMCCDA effectively predicted circRNA-disease associations, identifying 29 top candidates linked to BC in related studies (He et al. 2021a, b).

Moreover, circRNAs identified by a single method tend to include more false positives, suggesting that circRNAs detected by multiple tools are more dependable. CirComPara2 is a computational tool designed to aggregate expression estimates from various circRNA detection methods into a single, unified value. It does this by eliminating redundant counts of back-splicing junctions (BSJs) detected by multiple tools, without requiring additional read re-alignment. CirComPara2 incorporates nine different circRNA detection methods and examines circRNAs that might have been missed by one or more tools (potential false negatives). Interestingly, only 4% of circRNAs in the false-negative set were missed by all methods, while 96% were detected by at least one of the nine tools (Gaffo et al. 2022). This indicates that selecting circRNAs predicted by at least two methods can lead to more accurate results (Vromman et al. 2023).

## Challenges and future perspectives of circrnas in breast cancer: from pathogenesis to therapeutic potential

Despite the growing recognition of circRNAs as key regulators in BC pathogenesis, several challenges hinder their full clinical translation. Understanding the intricate roles of circRNAs in gene regulation, splicing, and cellular pathways remains complex, especially given their diverse and overlapping functions. While numerous circRNAs exhibit dysregulated expressions in BC, the precise mechanisms driving these alterations and their functional consequences in tumor suppression or promotion are not yet fully elucidated.

High-throughput sequencing and bioinformatics analyses have significantly advanced circRNA research, identifying multiple circRNA–miRNA–mRNA regulatory axes involved in BC development. Notably, several circRNA/miRNA axes, including circIFI30/miR-520b-3p, CircCDYL/miR-1275, and circ-DNMT1/miR-1236-3p, have been implicated in BC progression and therapy resistance (Du et al. 2018; Liang et al. 2020; Liu et al. 2020; Shi et al. 2024; Wang et al. 2023a, b; Xing et al. 2020; Xu et al. 2019). These interactions highlight the significance of these interactions in BC development and their potential as therapeutic targets.

Beyond their Fundamental roles in tumor biology, circRNAs are also implicated in therapy resistance. Approximately 30% of BC patients exhibit resistance to treatment, with TNBC showing a particularly poor response (Ortega et al. 2021; Reitz et al. 2021). CircRNAs such as hsa_circ_0006528, hsa_circ_0001839, and circ_0085495 have been linked to altered drug sensitivity, suggesting their potential as therapeutic targets (De Palma et al. 2022). This underscores the promising role of circRNAs in improving BC management by modulating treatment responses. However, further research is required to determine whether circRNA-based interventions could effectively counteract therapy resistance across different BC subtypes.

Future studies should prioritize interdisciplinary approaches integrating circRNA analysis with genomics, transcriptomics, proteomics, and metabolomics to uncover the broader regulatory networks influencing BC. Advanced preclinical models, such as patient-derived xenografts and organoids, will be instrumental in assessing circRNA function across BC subtypes and exploring their roles in chemoresistance. Additionally, the role of circRNAs in immune regulation and metabolic reprogramming warrants further exploration, particularly in the context of tumor microenvironment interactions.

From a clinical perspective, circRNAs hold immense promise as biomarkers for non-invasive BC detection, given their high stability in body fluids and exosomes. Exosomal circRNAs, in particular, offer a novel avenue for early diagnosis and personalized therapy, as they are protected from degradation and can effectively modulate gene expression in recipient cells (Hussen et al. 2023; Kumar et al. 2022). However, while many studies have demonstrated the potential diagnostic and prognostic value of circRNAs, large-scale clinical validation is still required before they can be integrated into routine clinical practice.

Therapeutic strategies targeting circRNAs remain in their infancy. Approaches such as synthetic inhibitors for oncogenic circRNAs and overexpression of tumor-suppressive circRNAs present promising avenues for BC treatment. However, most studies have been limited to preclinical models, and the feasibility of translating these findings into effective therapies remains uncertain. Nevertheless, optimizing delivery systems for circRNA-modulating compounds and ensuring their specificity in targeting BC cells are critical challenges that must be addressed before clinical implementation. Large-scale clinical trials are crucial to evaluate the efficacy, safety, and long-term benefits of circRNA-targeted therapies in BC patients.

To surpass the existing limitations in circRNA therapy, genome-scale RNA-targeting CRISPR/Cas13d screens have been incredibly potent. Zhang et al. (2021) conducted a CRISPR/Cas13d screen in hepatocellular carcinoma cells and uncovered circRNAs whose knockdown sensitized cells to sorafenib, illustrating that Cas13d libraries can target thousands of circRNAs with higher specificity than shRNA methods. Expanding on this, Wang et al. (2022a) employed triple-negative breast cancer patient-derived organoids and xenografts to demonstrate that circCREIT overexpression (through vector delivery) re-sensitized doxorubicin-resistant tumors to chemotherapy, illustrating how 3D and in vivo models can confirm functional circRNA targets in BC. Furthermore, nanoparticles based on lipid and polymer that deliver siRNAs targeting oncogenic circDnmt1 have effectively inhibited breast tumor growth in mouse models, illustrating feasible delivery methods for circRNA suppression (A. T. He et al. 2021a, b). Candidate prioritization is Further enabled by large-scale Functional screens and computational pipelines. An shRNA-based screen in prostate cancer cells identified 171 circRNAs required for proliferation, the majority independent of their linear isoforms, offering a roadmap for similar BC screens (A. T. He et al. 2021a, b). Additionally, one study demonstrated that transformer-based models and convolutional neural networks can effectively predict circRNA–miRNA and circRNA–protein interactions, quickly proposing regulatory axes for experimental validation (Zhao and Wang 2025). Combining these AI-powered predictions with CRISPR/Cas13d assays, organoid/patient-derived xenograft (PDX) validation, and nanoparticle delivery will establish a powerful pipeline to test and translate circRNA-based therapies in breast cancer.

In conclusion, while circRNAs represent a promising frontier in BC diagnosis, prognosis, and treatment, addressing existing challenges related to standardization, mechanistic insights, and clinical validation is crucial for their successful translation into clinical practice. Future advancements in genomics, precision medicine, and interdisciplinary research will be instrumental in harnessing the full therapeutic potential of circRNAs. Additionally, circRNA-targeted strategies may further enhance therapeutic outcomes, paving the way for circRNA-driven personalized medicine in BC management.

## Data Availability

No datasets were generated or analysed during the current study.

## References

[CR1] Abdelhamid AM, Zeinelabdeen Y, Manie T, Khallaf E, Assal RA, Youness RA (2024) miR-17-5p/STAT3/H19: A novel regulatory axis tuning ULBP2 expression in young breast cancer patients. Pathol Res Pract 263:155638. 10.1016/j.prp.2024.155638 39388743 10.1016/j.prp.2024.155638

[CR2] Abtin M, Nafisi N, Hosseinzadeh A, Kadkhoda S, Omranipour R, Sahebi L, Razipour M, Ghafouri-Fard S, Shakoori A (2024) Inhibition of breast cancer cell growth and migration through siRNA-mediated modulation of circ_0009910/miR-145-5p/MUC1 axis. Non-coding RNA Res 9(2):367–375. 10.1016/j.ncrna.2024.01.016 10.1016/j.ncrna.2024.01.016 PMC10950563 38511058

[CR3] Afzal S, Hassan M, Ullah S, Abbas H, Tawakkal F, Khan MA (2022) Breast cancer; discovery of novel diagnostic biomarkers, drug resistance, and therapeutic implications. Front Mol Biosci 9:783450. 10.3389/FMOLB.2022.783450 35265667 10.3389/fmolb.2022.783450 PMC8899313

[CR4] Almouh M, Razmara E, Bitaraf A, Ghazimoradi MH, Hassan ZM, Babashah S (2022) Circular RNAs play roles in regulatory networks of cell signaling pathways in human cancers. Life Sci 309:120975. 10.1016/j.lfs.2022.120975 36126723 10.1016/j.lfs.2022.120975

[CR5] Ameli-Mojarad M, Ameli-Mojarad M, Nourbakhsh M, Nazemalhosseini-Mojarad E (2021) Circular RNA hsa_circ_0005046 and hsa_circ_0001791 May become diagnostic biomarkers for breast cancer early detection. J Oncol 2021(2303946). 10.1155/2021/2303946 10.1155/2021/2303946 PMC8233096 34239561

[CR6] Ashrafizadeh M, Dai J, Torabian P, Nabavi N, Aref AR, Aljabali AAA, Tambuwala M, Zhu M (2024) Circular RNAs in EMT-driven metastasis regulation: modulation of cancer cell plasticity, tumorigenesis and therapy resistance. Cell Mol Life Sci 81(1):214. 10.1007/S00018-024-05236-W 38733529 10.1007/s00018-024-05236-w PMC11088560

[CR7] Asim MN, Ibrahim MA, Malik MI, Dengel A, Ahmed S (2022) Circ-locnet: a computational framework for circular RNA sub-cellular localization prediction. Int J Mol Sci 23(15):8221. 10.3390/IJMS23158221 35897818 10.3390/ijms23158221 PMC9329987

[CR8] Assal RA, Rashwan HH, Zakaria ZI, Sweillam JH, Fouda YM, Abdelhamid AM, Youness RA (2025) Deciphering the mysteries of MEG3 LncRNA and its implications in genitourinary cancers. Front Oncol 15(April):6–10. 10.3389/fonc.2025.1519103 10.3389/fonc.2025.1519103 PMC12000830 40242248

[CR9] Awata D, Radhakrishnan V, Shaath H, Elango R, Rashid S, Akhtar M, Azis TKA, Ahmed I, Ouararhni K, Al-Shabeeb Akil AS, Alajez NM (2025) Circular RNA profiling identifies circ_0001522, circ_0001278, and circ_0001801 as predictors of unfavorable prognosis and drivers of triple-negative breast cancer hallmarks. Cell Death Discov. 10.1038/S41420-025-02576-9 40634303 10.1038/s41420-025-02576-9 PMC12241340

[CR10] Beilerli A, Gareev I, Beylerli O, Yang G, Pavlov V, Aliev G, Ahmad A (2022) Circular rnas as biomarkers and therapeutic targets in cancer. Semin Cancer Biol 83:242–252. 10.1016/j.semcancer.2020.12.026 33434640 10.1016/j.semcancer.2020.12.026

[CR11] Bhan A, Soleimani M, Mandal SS (2017) Long noncoding RNA and cancer: a new paradigm. Cancer Res 77(15):3965–3981. 10.1158/0008-5472.CAN-16-2634 28701486 10.1158/0008-5472.CAN-16-2634 PMC8330958

[CR12] Boopathy GTK, Hong W (2019) Role of Hippo Pathway-YAP/TAZ signaling in angiogenesis. Front Cell Dev Biology 7:49. 10.3389/FCELL.2019.00049 10.3389/fcell.2019.00049 PMC6468149 31024911

[CR13] Bose R, Ain R (2018) Regulation of transcription by circular RNAs. Adv Exp Med Biol 1087:81–94. 10.1007/978-981-13-1426-1_7 30259359 10.1007/978-981-13-1426-1_7

[CR14] Bray F, Laversanne M, Sung H, Ferlay J, Siegel RL, Soerjomataram I, Jemal A (2024) Global cancer statistics 2022: GLOBOCAN estimates of incidence and mortality worldwide for 36 cancers in 185 countries. CA Cancer J Clin 74(3):229–263. 10.3322/CAAC.21834 38572751 10.3322/caac.21834

[CR15] Burks HE, Matossian MD, Rhodes LV, Phamduy T, Elliott S, Buechlein A, Rusch DB, Miller DFB, Nephew KP, Chrisey D, Collins-Burow BM, Burow ME (2021) ZEB2 regulates endocrine therapy sensitivity and metastasis in luminal a breast cancer cells through a non-canonical mechanism. Breast Cancer Res Treat 189(1):25–37. 10.1007/S10549-021-06256-X 34231077 10.1007/s10549-021-06256-x

[CR16] Cai ZR, Hu Y, Liao K, Li H, Chen DL, Ju HQ (2023) Circular rnas: emerging regulators of glucose metabolism in cancer. Cancer Lett. 10.1016/j.canlet.2022.215978 10.1016/j.canlet.2022.215978 36283584

[CR17] Carriero S, Damha MJ (2003) Inhibition of pre-mRNA splicing by synthetic branched nucleic acids. Nucleic Acids Res 31(21):6157–6167. 10.1093/NAR/GKG824 14576302 10.1093/nar/gkg824 PMC275466

[CR18] Ceci M, Fazi F, Romano N (2021) The role of RNA-binding and ribosomal proteins as specific RNA translation regulators in cellular differentiation and carcinogenesis. Biochimica et Biophysica Acta (BBA). 10.1016/j.bbadis.2020.166046 10.1016/j.bbadis.2020.166046 33383105

[CR19] Cen Y, Chen L, Liu Z, Lin Q, Fang X, Yao H, Gong C (2023) Novel roles of RNA-binding proteins in drug resistance of breast cancer: from molecular biology to targeting therapeutics. Cell Death Discov. 10.1038/S41420-023-01352-X 10.1038/s41420-023-01352-x PMC9911762 36759501

[CR20] Chang R, Yu H, Li S, Pan J (2024) CircRNA hsa_circ_0003528/miR-215 is considered a potential target for predictive prognosis and therapy for triple-negative breast cancer. Mol Biol Rep. 10.1007/S11033-024-09808-8 39126511 10.1007/s11033-024-09808-8

[CR21] Chen LL (2020) The expanding regulatory mechanisms and cellular functions of circular RNAs. Nat Rev Mol Cell Biol 21(8):475–490. 10.1038/S41580-020-0243-Y 32366901 10.1038/s41580-020-0243-y

[CR22] Chen B, Wei W, Huang X, Xie X, Kong Y, Dai D, Yang L, Wang J, Tang H, Xie X (2018a) Circepsti1 as a prognostic marker and mediator of triple-negative breast cancer progression. Theranostics 8(14):4003–4015. 10.7150/THNO.24106 30083277 10.7150/thno.24106 PMC6071524

[CR23] Chen N, Zhao G, Yan X, Lv Z, Yin H, Zhang S, Song W, Li X, Li L, Du Z, Jia L, Zhou L, Li W, Hoffman AR, Hu JF, Cui J (2018b) A novel FLI1 exonic circular RNA promotes metastasis in breast cancer by coordinately regulating TET1 and DNMT1. Genome Biol. 10.1186/S13059-018-1594-Y 30537986 10.1186/s13059-018-1594-y PMC6290540

[CR24] Chen T, Wang X, Li C, Zhang H, Liu Y, Han D, Li Y, Li Z, Luo D, Zhang N, Zheng M, Chen B, Wang L, Zhao W, Yang Q (2021) CircHIF1A regulated by FUS accelerates triple-negative breast cancer progression by modulating NFIB expression and translocation. Oncogene 40(15):2756–2771. 10.1038/S41388-021-01739-Z 33714984 10.1038/s41388-021-01739-z

[CR25] Chen D, Zeng S, Qiu H, Yang M, Lin X, Lv X, Li P, Weng S, Kou S, Luo K, Liu Z, Yi Y, Liu H (2024a) Circ-FOXO3 inhibits triple-negative breast cancer growth and metastasis via regulating WHSC1-H3K36me2-Zeb2 axis. Cell Signal. 10.1016/j.cellsig.2024.111079 38341124 10.1016/j.cellsig.2024.111079

[CR26] Chen H, Wang X, Cheng H, Deng Y, Chen J, Wang B (2024b) CircRNA circRREB1 promotes tumorigenesis and progression of breast cancer by activating Erk1/2 signaling through interacting with GNB4. Heliyon. 10.1016/j.heliyon.2024.e28785 38617926 10.1016/j.heliyon.2024.e28785 PMC11015410

[CR27] Conlon EG, Manley JL (2017) RNA-binding proteins in neurodegeneration: mechanisms in aggregate. Genes Dev 31(15):1509–1528. 10.1101/GAD.304055.117 28912172 10.1101/gad.304055.117 PMC5630017

[CR28] Cui Y, Fan J, Shi W, Zhou Z (2022) Circ_0001667 knockdown blocks cancer progression and attenuates adriamycin resistance by depleting NCOA3 via releasing miR-4458 in breast cancer. Drug Dev Res 83(1):75–87. 10.1002/DDR.21845 34227151 10.1002/ddr.21845

[CR29] Das A, Sinha T, Shyamal S, Panda AC (2021) Emerging role of circular RNA–protein interactions. Non-coding RNA. 10.3390/NCRNA7030048 34449657 10.3390/ncrna7030048 PMC8395946

[CR30] Dawoud A, Ihab Zakaria Z, Hisham Rashwan H, Braoudaki M, Youness RA (2023) Circular rnas: new layer of complexity evading breast cancer heterogeneity. Non-Coding RNA Res 8(1):60–74. 10.1016/j.ncrna.2022.09.011 10.1016/j.ncrna.2022.09.011 PMC9637558 36380816

[CR31] Dawoud A, Elmasri RA, Mohamed AH, Mahmoud A, Rostom MM, Youness RA (2024) Involvement of circRNAs in regulating the new generation of cancer hallmarks: a special depiction on hepatocellular carcinoma. Crit Rev Oncol Hematol 196:104312 38428701 10.1016/j.critrevonc.2024.104312

[CR32] De Palma FDE, Salvatore F, Pol JG, Kroemer G, Maiuri MC (2022) Circular rnas as potential biomarkers in breast cancer. Biomedicines. 10.3390/BIOMEDICINES10030725 35327527 10.3390/biomedicines10030725 PMC8945016

[CR33] Doghish AS, Mahmoud A, Abd-Elmawla MA, Zaki MB, Aborehab NM, Hatawsh A, Radwan AF, Sayed GA, Moussa R, Abdel-Reheim MA, Mohammed OA, Elimam H (2025) Innovative perspectives on glioblastoma: the emerging role of long non-coding RNAs. Funct Integr Genomics. 10.1007/S10142-025-01557-6 39992471 10.1007/s10142-025-01557-6

[CR34] Du WW, Yang W, Chen Y, Wu ZK, Foster FS, Yang Z, Li X, Yang BB (2017) Foxo3 circular rna promotes cardiac senescence by modulating multiple factors associated with stress and senescence responses. Eur Heart J 38(18):1402–1412. 10.1093/EURHEARTJ/EHW001 26873092 10.1093/eurheartj/ehw001

[CR35] Du WW, Yang W, Li X, Awan FM, Yang Z, Fang L, Lyu J, Li F, Peng C, Krylov SN, Xie Y, Zhang Y, He C, Wu N, Zhang C, Sdiri M, Dong J, Ma J, Gao C, Yang BB (2018) A circular RNA circ-DNMT1 enhances breast cancer progression by activating autophagy. Oncogene 37(44):5829–5842. 10.1038/S41388-018-0369-Y 29973691 10.1038/s41388-018-0369-y

[CR36] Du WW, Yang W, Li X, Fang L, Wu N, Li F, Chen Y, He Q, Liu E, Yang Z, Awan FM, Liu M, Yang BB (2020) The circular RNA circSKA3 binds integrin β1 to induce invadopodium formation enhancing breast cancer invasion. Mol Ther 28(5):1287–1298. 10.1016/j.ymthe.2020.03.002 32229309 10.1016/j.ymthe.2020.03.002 PMC7210749

[CR37] Elemam NM, Youness RA, Abdelhamid AM, Talaat IM (2024) CAR-NK/CAR-T cells: emerging immunotherapy of cancer. 1–37. 10.1007/16833_2024_429

[CR38] Fan Y, Wang J, Jin W, Sun Y, Xu Y, Wang Y, Liang X, Su D (2021) Circnr3c2 promotes HRD1-mediated tumor-suppressive effect via sponging miR-513a-3p in triple-negative breast cancer. Mol Cancer. 10.1186/S12943-021-01321-X 33530981 10.1186/s12943-021-01321-x PMC7851937

[CR39] Fang L, Du WW, Lyu J, Dong J, Zhang C, Yang W, He A, Kwok YSS, Ma J, Wu N, Li F, Awan FM, He C, Yang BL, Peng C, Mackay HJ, Yee AJ, Yang BB (2018) Enhanced breast cancer progression by mutant p53 is inhibited by the circular RNA circ-Ccnb1. Cell Death Differ 25(12):2195–2208. 10.1038/S41418-018-0115-6 29795334 10.1038/s41418-018-0115-6 PMC6261950

[CR40] Fontemaggi G, Turco C, Esposito G, Di Agostino S (2021) New molecular mechanisms and clinical impact of circrnas in human cancer. Cancers. 10.3390/CANCERS13133154 34202482 10.3390/cancers13133154 PMC8268751

[CR41] Fu B, Liu W, Zhu C, Li P, Wang L, Pan L, Li K, Cai P, Meng M, Wang Y, Zhang A, Tang W, An M (2021) Circular rna circbcbm1 promotes breast cancer brain metastasis by modulating mir-125a/brd4 axis. Int J Biol Sci 17(12):3104–3117. 10.7150/IJBS.58916 34421353 10.7150/ijbs.58916 PMC8375234

[CR42] Gaffo E, Buratin A, Dal Molin A, Bortoluzzi S (2022) Sensitive, reliable and robust circRNA detection from RNA-seq with CirComPara2. Brief Bioinform. 10.1093/BIB/BBAB418 34698333 10.1093/bib/bbab418 PMC8769706

[CR43] Galeano WC, Ramírez-Malule H, Gómez-Rios D (2024) Anticancer activity of monastrol, hybrids and derivatives: a comprehensive bibliometric analysis of recent research. J Appl Pharm Sci 14(2):073–082. 10.7324/JAPS.2024.152544

[CR44] Gao D, Qi X, Zhang X, Fang K, Guo Z, Li L (2019) Hsa_circRNA_0006528 as a competing endogenous RNA promotes human breast cancer progression by sponging miR-7-5p and activating the MAPK/ERK signaling pathway. Mol Carcinog 58(4):554–564. 10.1002/MC.22950 30520151 10.1002/mc.22950

[CR45] García-Muse T, Aguilera A (2019) R loops: from physiological to pathological roles. Cell 179(3):604–618. 10.1016/j.cell.2019.08.055 31607512 10.1016/j.cell.2019.08.055

[CR46] Ge E, Yang Y, Gang M, Fan C, Zhao Q (2020) Predicting human disease-associated circrnas based on locality-constrained linear coding. Genomics 112(2):1335–1342. 10.1016/J.YGENO.2019.08.001 31394170 10.1016/j.ygeno.2019.08.001

[CR47] Ghazimoradi MH, Babashah S (2022) The role of circRNA/miRNA/mRNA axis in breast cancer drug resistance. Front Oncol. 10.3389/FONC.2022.966083 36132137 10.3389/fonc.2022.966083 PMC9484461

[CR48] Granados-Principal S, Quiles JL, Ramirez-Tortosa CL, Sanchez-Rovira P, Ramirez-Tortosa M (2010) New advances in molecular mechanisms and the prevention of adriamycin toxicity by antioxidant nutrients. Food Chem Toxicol 48(6):1425–1438. 10.1016/j.fct.2010.04.007 20385199 10.1016/j.fct.2010.04.007

[CR49] Guo Q, Wang T, Yang Y, Gao L, Zhao Q, Zhang W, Xi T, Zheng L (2020) Transcriptional factor Yin Yang 1 promotes the stemness of breast cancer cells by suppressing miR-873-5p transcriptional activity. Mol Ther Nucleic Acids 21:527–541. 10.1016/j.omtn.2020.06.018 32711380 10.1016/j.omtn.2020.06.018 PMC7381513

[CR50] Hamburger AW (2008) The role of ErbB3 and its binding partners in breast cancer progression and resistance to hormone and tyrosine kinase directed therapies. J Mammary Gland Biol Neoplasia 13(2):225–233. 10.1007/S10911-008-9077-5 18425425 10.1007/s10911-008-9077-5 PMC3709461

[CR51] He R, Liu P, Xie X, Zhou Y, Liao Q, Xiong W, Li X, Li G, Zeng Z, Tang H (2017) CircGFRA1 and GFRA1 act as CeRNAs in triple negative breast cancer by regulating miR-34a. J Exp Clin Cancer Res 36(1):145. 10.1186/S13046-017-0614-1 29037220 10.1186/s13046-017-0614-1 PMC5644184

[CR52] He AT, Liu J, Li F, Yang BB (2021a) Targeting circular RNAs as a therapeutic approach: current strategies and challenges. Signal Transduct Target Ther 6(1):185. 10.1038/S41392-021-00569-5 34016945 10.1038/s41392-021-00569-5 PMC8137869

[CR53] He X, Xu T, Hu W, Tan Y, Wang D, Wang Y, Zhao C, Yi Y, Xiong M, Lv W, Wu M, Li X, Wu Y, Zhang Q (2021b) Circular rnas: their role in the pathogenesis and orchestration of breast cancer. Front Cell Dev Biol. 10.3389/FCELL.2021.647736 33777954 10.3389/fcell.2021.647736 PMC7991790

[CR54] He M, Pan Y, You C, Gao H (2024) Circrnas in cancer therapy tolerance. Clin Chim Acta. 10.1016/j.cca.2024.119684 38649011 10.1016/j.cca.2024.119684

[CR55] Hu K, Liu X, Li Y, Li Q, Xu Y, Zeng W, Zhong G, Yu C (2020a) Exosomes mediated transfer of Circ-UBE2D2 enhances the resistance of breast cancer to Tamoxifen by binding to MiR-200a-3p. Med Sci Monit 26. 10.12659/MSM.922253 10.12659/MSM.922253 PMC7431386 32756532

[CR56] Hu Y, Song Q, Zhao J, Ruan J, He F, Yang X, Yu X (2020b) Identification of plasma hsa_circ_0008673 expression as a potential biomarker and tumor regulator of breast cancer. J Clin Lab Anal. 10.1002/JCLA.23393 32808350 10.1002/jcla.23393 PMC7521290

[CR57] Huang Y, Zhu Q (2021) Mechanisms regulating abnormal circular RNA biogenesis in cancer. Cancers. 10.3390/CANCERS13164185 34439339 10.3390/cancers13164185 PMC8391333

[CR58] Huang A, Zheng H, Wu Z, Chen M, Huang Y (2020) Circular RNA-protein interactions: functions, mechanisms, and identification. Theranostics 10(8):3506–3517. 10.7150/THNO.42174 10.7150/thno.42174 PMC7069073 32206104

[CR59] Huang Q, He Y, Zhang X, Guo L (2021a) Circular Rna hsa_circ_0103552 promotes proliferation, migration, and invasion of breast cancer cells through upregulating cysteine-rich angiogenic inducer 61 (Cyr61) expression via sponging microrna-515-5p. Tohoku J Exp Med 255(2):171–181. 10.1620/TJEM.255.171 34707022 10.1620/tjem.255.171

[CR60] Huang Y, Zheng W, Ji C, Wang X, Yu Y, Deng X, Zhou X, Fang L (2021b) Circular RNA circRPPH1 promotes breast cancer progression via circRPPH1-miR-512-5p-STAT1 axis. Cell Death Discov. 10.1038/S41420-021-00771-Y 34873163 10.1038/s41420-021-00771-y PMC8648777

[CR61] Huang L, Zhang G, Han L, Bai X, Xi Z, Wang F, Han G (2024) Circ_0059457 promotes proliferation, metastasis, sphere formation and glycolysis in breast cancer cells by sponging miR-140-3p to regulate UBE2C. Biochem Genet 62(1):125–143. 10.1007/S10528-023-10407-8 37284894 10.1007/s10528-023-10407-8

[CR62] Hussen BM, Mohamadtahr S, Abdullah SR, Hidayat HJ, Rasul MF, Faraj H, Ghafouri-Fard GS, Taheri S, Khayamzadeh M, M., Jamali E (2023) Exosomal circular RNAs: New player in breast cancer progression and therapeutic targets. Front Genet 14. 10.3389/FGENE.2023.1126944 10.3389/fgene.2023.1126944 PMC10011470 36926585

[CR63] Javid H, Hashemian P, Yazdani S, Sharbaf Mashhad A, Karimi-Shahri M (2022) The role of heat shock proteins in metastatic colorectal cancer: a review. J Cell Biochem 123(11):1704–1735. 10.1002/JCB.30326 36063530 10.1002/jcb.30326

[CR64] Jeck WR, Sharpless NE (2014) Detecting and characterizing circular RNAs. Nat Biotechnol 32(5):453–461. 10.1038/NBT.2890 24811520 10.1038/nbt.2890 PMC4121655

[CR65] Jiang MP, Xu WX, Hou JC, Xu Q, Wang DD, Tang JH (2021) The emerging role of the interactions between circular RNAs and RNA-binding proteins in common human cancers. J Cancer 12(17):5206–5219. 10.7150/JCA.58182 34335937 10.7150/jca.58182 PMC8317540

[CR66] Kciuk M, Gielecińska A, Budzinska A, Mojzych M, Kontek R (2022) Metastasis and MAPK pathways. Int J Mol Sci. 10.3390/IJMS23073847 35409206 10.3390/ijms23073847 PMC8998814

[CR67] Korpal M, Lee ES, Hu G, Kang Y (2008) The miR-200 family inhibits epithelial-mesenchymal transition and cancer cell migration by direct targeting of E-cadherin transcriptional repressors ZEB1 and ZEB2. J Biol Chem 283(22):14910–14914. 10.1074/jbc.C800074200 18411277 10.1074/jbc.C800074200 PMC3258899

[CR68] Kumar DN, Chaudhuri A, Aqil F, Dehari D, Munagala R, Singh S, Gupta RC, Agrawal AK (2022) Exosomes as emerging drug delivery and diagnostic modality for breast cancer: recent advances in isolation and application. Cancers. 10.3390/CANCERS14061435 35326585 10.3390/cancers14061435 PMC8946254

[CR69] Lan J, Wang L, Cao J, Wan Y, Zhou Y (2024) Circbraf promotes the progression of triple-negative breast cancer through modulating methylation by recruiting KDM4B to histone H3K9me3 and IGF2BP3 to mrna. Am J Cancer Res 14(5):2020–2036. 10.62347/OOLG5765 38859856 10.62347/OOLG5765 PMC11162659

[CR70] Lei M, Zheng G, Ning Q, Zheng J, Dong D (2020) Translation and functional roles of circular RNAs in human cancer. Mol Cancer. 10.1186/S12943-020-1135-7 32059672 10.1186/s12943-020-1135-7 PMC7023758

[CR71] Li Z, Huang C, Bao C, Chen L, Lin M, Wang X, Zhong G, Yu B, Hu W, Dai L, Zhu P, Chang Z, Wu Q, Zhao Y, Jia Y, Xu P, Liu H, Shan G (2015) Exon-intron circular RNAs regulate transcription in the nucleus. Nat Struct Mol Biol 22(3):256–264. 10.1038/NSMB.2959 25664725 10.1038/nsmb.2959

[CR72] Li X, Yang L, Chen LL (2018) The biogenesis, functions, and challenges of circular RNAs. Mol Cell 71(3):428–442. 10.1016/j.molcel.2018.06.034 30057200 10.1016/j.molcel.2018.06.034

[CR73] Li D, Yang Y, Li ZQ, Li LC, Zhu XH (2019) Circular rnas: from biogenesis and function to diseases. Chin Med J 132(20):2457–2464. 10.1097/CM9.0000000000000465 31651510 10.1097/CM9.0000000000000465 PMC6831080

[CR74] Li J, Tang Q, Dong W, Wang Y (2020a) Circbach1/let-7a-5p axis enhances the proliferation and metastasis of colorectal cancer by upregulating CREB5 expression. J Gastrointest Oncol 11(6):1186–1199. 10.21037/JGO-20-498 33456992 10.21037/jgo-20-498 PMC7807282

[CR75] Li Z, Chen Z, Feng Y, Hu G, Jiang Y (2020b) CircMMP11 acts as a ce-circRNA in breast cancer progression by regulating miR-1204. Am J Transl Res 12(6):2585 32655792 PMC7344057

[CR76] Li H, Li Q, He S (2021) Hsa_circ_0025202 suppresses cell tumorigenesis and Tamoxifen resistance via miR-197-3p/HIPK3 axis in breast cancer. World J Surg Oncol. 10.1186/S12957-021-02149-X 33536026 10.1186/s12957-021-02149-x PMC7860040

[CR77] Li Y, Wang Z, Su P, Liang Y, Li Z, Zhang H, Song X, Han D, Wang X, Liu Y, Yang J, Chen B, Wang L, Zhao W, Yang Q (2022) circ-EIF6 encodes EIF6-224aa to promote TNBC progression via stabilizing MYH9 and activating the Wnt/beta-catenin pathway. Mol Ther 30(1):415–430. 10.1016/j.ymthe.2021.08.026 34450253 10.1016/j.ymthe.2021.08.026 PMC8753373

[CR78] Li J, Dong X, Kong X, Wang Y, Li Y, Tong Y, Zhao W, Duan W, Li P, Wang Y, Wang C (2023a) Circular RNA hsa_circ_0067842 facilitates tumor metastasis and immune escape in breast cancer through HuR/CMTM6/PD-L1 axis. Biol Direct. 10.1186/S13062-023-00397-3 37592296 10.1186/s13062-023-00397-3 PMC10436663

[CR79] Li J, Han Y, Wang S, Wu X, Cao J, Sun T (2023b) Circular rnas: biogenesis, biological functions, and roles in myocardial infarction. Int J Mol Sci. 10.3390/IJMS24044233 36835653 10.3390/ijms24044233 PMC9963350

[CR80] Li S, Zeng H, Fan J, Wang F, Xu C, Li Y, Tu J, Nephew KP, Long X (2023c) Glutamine metabolism in breast cancer and possible therapeutic targets. Biochem Pharmacol 210:115464. 10.1016/j.bcp.2023.115464 36849062 10.1016/j.bcp.2023.115464

[CR81] Li Z, Li Y, Han D, Wang X, Li C, Chen T, Li W, Liang Y, Luo D, Chen B, Wang L, Zhao W, Yang Q (2023d) Circrna-SFMBT2 orchestrates ERα activation to drive tamoxifen resistance in breast cancer cells. Cell Death Dis. 10.1038/S41419-023-06006-5 37524698 10.1038/s41419-023-06006-5 PMC10390580

[CR82] Liang HF, Zhang XZ, Liu BG, Jia GT, Li WL (2017) Circular RNA circ-ABCB10 promotes breast cancer proliferation and progression through sponging miR-1271. Am J Cancer Res 7(7):1566 28744405 PMC5523036

[CR83] Liang Y, Song X, Li Y, Ma T, Su P, Guo R, Chen B, Zhang H, Sang Y, Liu Y, Duan Y, Zhang N, Li X, Zhao W, Wang L, Yang Q (2019a) Targeting the circBMPR2/miR-553/USP4 axis as a potent therapeutic approach for breast cancer. Mol Therapy Nucleic Acids 17:347–361. 10.1016/j.omtn.2019.05.005 31302495 10.1016/j.omtn.2019.05.005 PMC6626870

[CR84] Liang Y, Song X, Li Y, Ma T, Su P, Guo R, Chen B, Zhang H, Sang Y, Liu Y, Duan Y, Zhang N, Li X, Zhao W, Wang L, Yang Q (2019b) Targeting the circBMPR2/miR-553/USP4 axis as a potent therapeutic approach for breast cancer. Mol Therapy Nucleic Acids 17(September):347–361. 10.1016/j.omtn.2019.05.005 31302495 10.1016/j.omtn.2019.05.005 PMC6626870

[CR85] Liang G, Ling Y, Mehrpour M, Saw PE, Liu Z, Tan W, Tian Z, Zhong W, Lin W, Luo Q, Lin Q, Li Q, Zhou Y, Hamai A, Codogno P, Li J, Song E, Gong C (2020) Autophagy-associated circRNA circCDYL augments autophagy and promotes breast cancer progression. Mol Cancer. 10.1186/S12943-020-01152-2 32213200 10.1186/s12943-020-01152-2 PMC7093993

[CR86] Liang R, Zhang J, Liu Z, Liu Z, Li Q, Luo X, Li Y, Ye J, Lin Y (2021a) Mechanism and molecular network of RBM8A-mediated regulation of oxaliplatin resistance in hepatocellular carcinoma. Front Oncol. 10.3389/FONC.2020.585452 33552961 10.3389/fonc.2020.585452 PMC7862710

[CR87] Liang Y, Song X, Li Y, Su P, Han D, Ma T, Guo R, Chen B, Zhao W, Sang Y, Zhang N, Li X, Zhang H, Liu Y, Duan Y, Wang L, Yang Q (2021b) Correction: circKDM4C suppresses tumor progression and attenuates doxorubicin resistance by regulating miR-548p/PBLD axis in breast cancer (Oncogene, (2019), 38, 42, (6850–6866). 10.1038/s41388-019-0926-z 10.1038/s41388-019-0926-z 31406252

[CR88] Liang L, Gao M, Li W, Tang J, He Q, Zeng F, Cao J, Liu S, Chen Y, Li X, Zhou Y (2024a) CircGSK3β mediates PD-L1 transcription through miR-338-3p/PRMT5/H3K4me3 to promote breast cancer cell immune evasion and tumor progression. Cell Death Discov. 10.1038/S41420-024-02197-8 39366935 10.1038/s41420-024-02197-8 PMC11452702

[CR89] Liang Y, Ye F, Luo D, Long L, Wang Y, Jin Y, Wang L, Li Y, Han D, Chen B, Zhao W, Wang L, Yang Q (2024b) Exosomal circSIPA1L3-mediated intercellular communication contributes to glucose metabolic reprogramming and progression of triple negative breast cancer. Mol Cancer 23(1):125. 10.1186/S12943-024-02037-4 38849860 10.1186/s12943-024-02037-4 PMC11161950

[CR90] Liu B, Sun L, Liu Q, Gong C, Yao Y, Lv X, Lin L, Yao H, Su F, Li D, Zeng M, Song E (2015) A cytoplasmic NF-κB interacting long noncoding RNA blocks IκB phosphorylation and suppresses breast cancer metastasis. Cancer Cell 27(3):370–381. 10.1016/j.ccell.2015.02.004 25759022 10.1016/j.ccell.2015.02.004

[CR91] Liu Y, Dong Y, Zhao L, Su L, Luo J (2018) Circular *RNAMTO1* suppresses breast cancer cell viability and reverses monastrol resistance through regulating the TRAF4/Eg5 axis. Int J Oncol 53(4):1752–1762. 10.3892/IJO.2018.4485 30015883 10.3892/ijo.2018.4485

[CR92] Liu M, Wang Q, Shen J, Yang BB, Ding X (2019) Circbank: a comprehensive database for circrna with standard nomenclature. RNA Biol 16(7):899–905. 10.1080/15476286.2019.1600395 31023147 10.1080/15476286.2019.1600395 PMC6546381

[CR93] Liu G, Zhang Z, Song Q, Guo Y, Bao P, Shui H (2020) Circ_0006528 contributes to Paclitaxel resistance of breast cancer cells by regulating mir-1299/cdk8 axis. Onco Targets Ther 13:9497–9511. 10.2147/OTT.S252886 33061434 10.2147/OTT.S252886 PMC7522311

[CR94] Liu C, Chen M, Shi Y (2021a) Downregulation of hsa_circ_0006220 and its correlation with clinicopathological factors in human breast cancer. Gland Surg 10(1):816–825. 10.21037/GS-21-42 33708563 10.21037/gs-21-42 PMC7944078

[CR95] Liu J, Peng X, Liu Y, Hao R, Zhao R, Zhang L, Zhao F, Liu Q, Liu Y, Qi Y (2021b) The diagnostic value of serum exosomal has_circ_0000615 for breast cancer patients. Int J Gen Med. 10.2147/IJGM.S319801 34429639 10.2147/IJGM.S319801 PMC8379395

[CR96] Liu W, Xiong Y, Wan R, Shan R, Li J, Wen W (2021c) The roles of circMTO1 in cancer. Front Cell Dev Biol. 10.3389/FCELL.2021.656258 34277605 10.3389/fcell.2021.656258 PMC8277961

[CR97] Liu X, Zhang Y, Zhou S, Dain L, Mei L, Zhu G (2022) Circular RNA: an emerging frontier in RNA therapeutic targets, RNA therapeutics, and mRNA vaccines. J Controlled Release 348:84–94. 10.1016/J.JCONREL.2022.05.043 10.1016/j.jconrel.2022.05.043 PMC9644292 35649485

[CR98] Liu W, Zhang J, Zhang J, Ye Y, Zhu J, Yu Q, Li T, Sun X, Chen H (2024) EIF4A3-induced circ_0022382 promotes breast cancer cell progression through the let-7a-5p/PI3K/AKT/mTOR signaling pathway and SLC7A11 axis. Front Oncol 14. 10.3389/FONC.2024.1476731 10.3389/fonc.2024.1476731 PMC11758171 39868374

[CR99] Lu ZN, Song J, Sun TH, Sun G (2021) UBE2C affects breast cancer proliferation through the akt/mtor signaling pathway. Chin Med J 134(20):2465–2474. 10.1097/CM9.0000000000001708 34620747 10.1097/CM9.0000000000001708 PMC8654430

[CR100] Lugano R, Ramachandran M, Dimberg A (2020) Tumor angiogenesis: causes, consequences, challenges and opportunities. Cell Mol Life Sci 77(9):1745–1770. 10.1007/S00018-019-03351-7 31690961 10.1007/s00018-019-03351-7 PMC7190605

[CR101] Łukasiewicz S, Czeczelewski M, Forma A, Baj J, Sitarz R, Stanisławek A (2021) Breast cancer—epidemiology, risk factors, classification, prognostic markers, and current treatment strategies—an updated review. Cancers (Basel). 10.3390/CANCERS13174287 34503097 10.3390/cancers13174287 PMC8428369

[CR102] Luo N, Liu S, Li X, Hu Y, Zhang K (2021) Circular RNA circHIPK3 promotes breast cancer progression via sponging miR-326. Cell Cycle 20(13):1320–1333. 10.1080/15384101.2021.1939476 34152928 10.1080/15384101.2021.1939476 PMC8331001

[CR103] Lyu L, Zhang S, Deng Y, Wang M, Deng X, Yang S, Wu Y, Dai Z (2021) Regulatory mechanisms, functions, and clinical significance of circRNAs in triple-negative breast cancer. J Hematol Oncol. 10.1186/S13045-021-01052-Y 33676555 10.1186/s13045-021-01052-y PMC7937293

[CR104] Maadi H, Soheilifar MH, Choi WS, Moshtaghian A, Wang Z (2021) Trastuzumab mechanism of action; 20 years of research to unravel a dilemma. Cancers. 10.3390/CANCERS13143540 34298754 10.3390/cancers13143540 PMC8303665

[CR105] Malmgren JA, Mayer M, Atwood MK, Kaplan HG (2018) Differential presentation and survival of de novo and recurrent metastatic breast cancer over time: 1990–2010. Breast Cancer Res Treat 167(2):579–590. 10.1007/S10549-017-4529-5 29039120 10.1007/s10549-017-4529-5 PMC5790843

[CR106] Manni W, Jianxin X, Weiqi H, Siyuan C, Huashan S (2022) JMJD family proteins in cancer and inflammation. Signal Transduct Target Therapy 7(1). 10.1038/S41392-022-01145-1 10.1038/s41392-022-01145-1 PMC9434538 36050314

[CR107] Meng S, Zhou H, Feng Z, Xu Z, Tang Y, Li P, Wu M (2017) CircRNA: functions and properties of a novel potential biomarker for cancer. Mol Cancer. 10.1186/S12943-017-0663-2 28535767 10.1186/s12943-017-0663-2 PMC5440908

[CR108] Misir S, Yaman SO, Petrović N, Sumer C, Hepokur C, Aliyazicioglu Y (2022) Circrnas in drug resistance of breast cancer. Oncol Res 30(4):157–172. 10.32604/OR.2022.027547 37304411 10.32604/or.2022.027547 PMC10208077

[CR109] Moghaddam MB, Maleki M, Oveisee M, Moghaddam MB, Arabian M, Malakootian M (2022) Circular rnas: new players in cardiomyopathy. Genes 13(9):1537. 10.3390/GENES13091537 36140705 10.3390/genes13091537 PMC9498503

[CR110] Mumtaz PT, Taban Q, Dar MA, Mir S, Haq ZU, Zargar SM, Shah RA, Ahmad SM (2020) Deep insights in circular rnas: from biogenesis to therapeutics. Biol Proced Online 22(1):10. 10.1186/S12575-020-00122-8 32467674 10.1186/s12575-020-00122-8 PMC7227217

[CR111] Neophytou CM, Trougakos IP, Erin N, Papageorgis P (2021) Apoptosis deregulation and the development of cancer multi-drug resistance. Cancers 13(17). 10.3390/CANCERS13174363 10.3390/cancers13174363 PMC8430856 34503172

[CR112] Nguyen DT, Trac QT, Nguyen TH, Nguyen HN, Ohad N, Pawitan Y, Vu TN (2021) Circall: fast and accurate methodology for discovery of circular RNAs from paired-end RNA-sequencing data. BMC Bioinformatics. 10.1186/S12859-021-04418-8 34645386 10.1186/s12859-021-04418-8 PMC8513298

[CR113] Nielsen AF, Bindereif A, Bozzoni I, Hanan M, Hansen TB, Irimia M, Kadener S, Kristensen LS, Legnini I, Morlando M, Jarlstad Olesen MT, Pasterkamp RJ, Preibisch S, Rajewsky N, Suenkel C, Kjems J (2022) Best practice standards for circular RNA research. Nat Methods 19(10):1208–1220. 10.1038/S41592-022-01487-2 35618955 10.1038/s41592-022-01487-2 PMC9759028

[CR114] Obeng E (2021) Apoptosis (Programmed cell death) and its signals-a review. Braz J Biol 81(4):1133–1143. 10.1590/1519-6984.228437 33111928 10.1590/1519-6984.228437

[CR115] Ortega MA, Fraile-Martínez O, García-Montero C, Pekarek L, Guijarro LG, Castellanos AJ, Sanchez-Trujillo L, García-Honduvilla N, Álvarez-Mon M, Buján J, Zapico Á, Lahera G, Álvarez-Mon MA (2021) Physical activity as an imperative support in breast cancer management. Cancers 13(1):1–30. 10.3390/CANCERS13010055 10.3390/cancers13010055 PMC7796347 33379177

[CR116] Palazzo AF, Koonin EV (2020) Functional long non-coding RNAs evolve from junk transcripts. Cell 183(5):1151–1161. 10.1016/j.cell.2020.09.047 33068526 10.1016/j.cell.2020.09.047

[CR117] Patop IL, Wüst S, Kadener S (2019) Past, present, and future of circRNAs. EMBO J 38(16):e100836. 10.15252/EMBJ.2018100836 31343080 10.15252/embj.2018100836 PMC6694216

[CR118] Pei X, Zhang Y, Wang X, Xue B, Sun M, Li H (2020) Circular RNA circ-ZEB1 acts as an oncogene in triple negative breast cancer via sponging miR-448. Int J Biochem Cell Biol. 10.1016/j.biocel.2020.105798 32629026 10.1016/j.biocel.2020.105798

[CR119] Pritchard JE, Dillon PM, Conaway MR, Silva CM, Parsons SJ (2012) A mechanistic study of the effect of doxorubicin/adriamycin on the estrogen response in a breast cancer model. Oncol (Switzerland) 83(6):305–320. 10.1159/000341394 10.1159/000341394 PMC3677554 22964943

[CR120] Rao AKDM, Arvinden VR, Ramasamy D, Patel K, Meenakumari B, Ramanathan P, Sundersingh S, Sridevi V, Rajkumar T, Herceg Z, Gowda H, Mani S (2021) Identification of novel dysregulated circular RNAs in early-stage breast cancer. J Cell Mol Med 25(8):3912–3921. 10.1111/JCMM.16324 33544410 10.1111/jcmm.16324 PMC8051735

[CR121] Rebolledo C, Silva JP, Saavedra N, Maracaja-Coutinho V (2023) Computational approaches for circrnas prediction and in Silico characterization. Brief Bioinform 24(3). 10.1093/BIB/BBAD154 10.1093/bib/bbad154 PMC10199773 37139555

[CR122] Reitz LK, Baptista SdeL, Santos EdaS, Hinnig PF, Rockenbach G, Vieira FGK, de Assis MAA, da Silva EL, Boaventura BCB, Di Pietro PF (2021) Diet quality is associated with serum antioxidant capacity in women with breast cancer: A cross sectional study. Nutrients 13(1):1–14. 10.3390/NU13010115 10.3390/nu13010115 PMC7824615 33396963

[CR123] Ren W, Yuan Y, Peng J, Mutti L, Jiang X (2022) The function and clinical implication of circular RNAs in lung cancer. Front Oncol. 10.3389/FONC.2022.862602 36338714 10.3389/fonc.2022.862602 PMC9629004

[CR124] Ren J, Chen W, Zhou Y, Sun J, Jiang G (2024) The novel circRNA circ_0045881 inhibits cell proliferation and invasion by targeting miR-214-3p in triple-negative breast cancer. BMC Cancer. 10.1186/S12885-024-12007-0 38429642 10.1186/s12885-024-12007-0 PMC10905830

[CR125] Robic A, Kühn C (2020) Beyond back splicing, a still poorly explored world: non-canonical circular RNAs. Genes 11(9):1–11. 10.3390/GENES11091111 10.3390/genes11091111 PMC7565381 32972011

[CR126] Rocca A, Braga L, Volpe MC, Maiocchi S, Generali D (2022) The predictive and prognostic role of RAS–RAF–MEK–ERK pathway alterations in breast cancer: revision of the literature and comparison with the analysis of cancer genomic datasets. Cancers. 10.3390/CANCERS14215306 36358725 10.3390/cancers14215306 PMC9653766

[CR127] Rostom MM, Rashwan AA, Sotiropoulou CD, Hozayen SZ, Abdelhamid AM, Abdelhalim MM, Eltahtawy O, Emara HM, Elemam NM, Kontos CK, Youness RA (2025) MIAT: A pivotal oncogenic long noncoding RNA tunning the hallmarks of solid malignancies. Transl Oncol 54. 10.1016/j.tranon.2025.102329 10.1016/j.tranon.2025.102329 PMC11910686 40014977

[CR128] Rybak-Wolf A, Stottmeister C, Glažar P, Jens M, Pino N, Giusti S, Hanan M, Behm M, Bartok O, Ashwal-Fluss R, Herzog M, Schreyer L, Papavasileiou P, Ivanov A, Öhman M, Refojo D, Kadener S, Rajewsky N (2014) Circular rnas in the mammalian brain are highly abundant, conserved, and dynamically expressed. Mol Cell 58(5):870–885. 10.1016/j.molcel.2015.03.027 10.1016/j.molcel.2015.03.027 25921068

[CR129] Sadlak J, Joshi I, Prószyński TJ, Kischel A (2023) CircAMOTL1 RNA and AMOTL1 protein: complex functions of AMOTL1 gene products. Int J Mol Sci. 10.3390/IJMS24032103 10.3390/ijms24032103 PMC9916871 36768425

[CR130] Sang Y, Chen B, Song X, Li Y, Liang Y, Han D, Zhang N, Zhang H, Liu Y, Chen T, Li C, Wang L, Zhao W, Yang Q (2019) Circrna_0025202 regulates Tamoxifen sensitivity and tumor progression via regulating the miR-182-5p/FOXO3a axis in breast cancer. Mol Ther 27(9):1638–1652. 10.1016/j.ymthe.2019.05.011 31153828 10.1016/j.ymthe.2019.05.011 PMC6731174

[CR131] Schiliro C, Firestein BL (2021) Mechanisms of metabolic reprogramming in cancer cells supporting enhanced growth and proliferation. Cells 10(5):1056. 10.3390/CELLS10051056 33946927 10.3390/cells10051056 PMC8146072

[CR132] Seal RL, Chen L, Griffiths-Jones S, Lowe TM, Mathews MB, O’Reilly D, Pierce AJ, Stadler PF, Ulitsky I, Wolin SL, Bruford EA (2020) A guide to naming human non‐coding RNA genes. EMBO J 39(6). 10.15252/EMBJ.2019103777 10.15252/embj.2019103777 PMC7073466 32090359

[CR133] Shagufta, Ahmad I (2018) Tamoxifen a pioneering drug: an update on the therapeutic potential of Tamoxifen derivatives. Eur J Med Chem 143:515–531. 10.1016/j.ejmech.2017.11.056 29207335 10.1016/j.ejmech.2017.11.056

[CR134] Sharpless WRJ, Norman E (2014) Detecting and characterizing circular RNAs. Nat Biotechnol 32(5):453–461. 10.1038/nbt.2890.Detecting 24811520 10.1038/nbt.2890 PMC4121655

[CR135] Shen B-J, Yang YF, Zhang XX (2023) Hsa_circ_0001925 promotes malignant progression in triple-negative breast cancer via miR-1299/YY1 axis. Thorac Cancer 14(8):746–757. 10.1111/1759-7714.14803 36754085 10.1111/1759-7714.14803 PMC10008682

[CR136] Shi X, Liao S, Bi Z, Liu J, Li H, Feng C (2023) Newly discovered circRNAs encoding proteins: recent progress. Front Genet. 10.3389/FGENE.2023.1264606 37829278 10.3389/fgene.2023.1264606 PMC10565661

[CR137] Shi G, Li H, Chen Y, Chen Z, Lin X (2024) Circsept9 promotes breast cancer progression by regulating PTBP3 expression via sponging miR-625-5p. Thorac Cancer 15(10):808–819. 10.1111/1759-7714.15252 38409914 10.1111/1759-7714.15252 PMC10995703

[CR138] Singh S, Sinha T, Panda AC (2024) Regulation of microrna by circular RNA. WIREs RNA. 10.1002/WRNA.1820 37783567 10.1002/wrna.1820

[CR139] Smid M, Wilting SM, Uhr K, Rodríguez-González FG, De Weerd V, Prager-Van Der Smissen WJC, Van Der Vlugt-Daane M, Van Galen A, Nik-Zainal S, Butler A, Martin S, Davies HR, Staaf J, Van De Vijver MJ, Richardson AL, MacGrogan G, Salgado R, Van Den Eynden GGGM, Purdie CA, Martens JWM (2019) The circular RNome of primary breast cancer. Genome Res 29(3):356–366. 10.1101/GR.238121.118 30692147 10.1101/gr.238121.118 PMC6396421

[CR140] Song X, Liang Y, Sang Y, Li Y, Zhang H, Chen B, Du L, Liu Y, Wang L, Zhao W, Ma T, Wang C, Yang Q (2020) Circhmcu promotes proliferation and metastasis of breast cancer by sponging the let-7 family. Mol Ther Nucleic Acids 20:518–533. 10.1016/J.OMTN.2020.03.014 32330870 10.1016/j.omtn.2020.03.014 PMC7178009

[CR141] Song X, Chen B, Liang Y, Li Y, Zhang H, Han D, Wang Y, Ye F, Wang L, Zhao W, Yang Q (2022) CircEIF3H-IGF2BP2-HuR scaffold complex promotes TNBC progression via stabilizing HSPD1/RBM8A/G3BP1 mRNA. Cell Death Discov. 10.1038/S41420-022-01055-9 35568705 10.1038/s41420-022-01055-9 PMC9107465

[CR142] Song R, Guo P, Ren X, Zhou L, Li P, Rahman NA, Wołczyński S, Li X, Zhang Y, Liu M, Liu J, Li X (2023) A novel polypeptide CAPG-171aa encoded by circcapg plays a critical role in triple-negative breast cancer. Mol Cancer 22(1):104. 10.1186/s12943-023-01806-x 37408008 10.1186/s12943-023-01806-x PMC10320902

[CR143] Stoyanov D, Stoyanov GS, Ivanov MN, Spasov RH, Tonchev AB (2023) Transcription factor Zbtb20 as a regulator of malignancy and its practical applications. Int J Mol Sci. 10.3390/IJMS241813763 37762065 10.3390/ijms241813763 PMC10530547

[CR144] Sun Z, Niu S, Xu F, Zhao W, Ma R, Chen M (2020) CircAMOTL1 promotes tumorigenesis through miR-526b/SIK2 axis in cervical cancer. Front Cell Dev Biol. 10.3389/FCELL.2020.568190 33344445 10.3389/fcell.2020.568190 PMC7744824

[CR145] Sun L, Chen S, Wang T, Bi S (2023) Hsa_circ_0008673 promotes breast cancer progression by MiR-578/GINS4 axis. Clin Breast Cancer 23(3):281–290. 10.1016/j.clbc.2022.12.015 36628810 10.1016/j.clbc.2022.12.015

[CR146] Tang H, Huang X, Wang J, Yang L, Kong Y, Gao G, Zhang L, Chen ZS, Xie X (2019) CircKIF4A acts as a prognostic factor and mediator to regulate the progression of triple-negative breast cancer. Mol Cancer. 10.1186/S12943-019-0946-X 30744636 10.1186/s12943-019-0946-x PMC6369546

[CR147] Tao X, Na L, Hu EX, Wang J, Wu LG, Zhang X, Wang L, Bin (2024) Clinical diagnostic value of circ-ARHGER28 for breast cancer and its effect on MCF 7 cell proliferation and apoptosis. Anticancer Res 44(7):2877–2886. 10.21873/ANTICANRES.17100 38925846 10.21873/anticanres.17100

[CR148] Tsai CN, Yu SC, Lee CW, Pang JHS, Wu CH, Lin SE, Chung YH, Tsai CL, Hsieh SY, Yu MC (2020) SOX4 activates CXCL12 in hepatocellular carcinoma cells to modulate endothelial cell migration and angiogenesis in vivo. Oncogene 39(24):4695–4710. 10.1038/S41388-020-1319-Z 32404985 10.1038/s41388-020-1319-z

[CR149] Turco C, Esposito G, Iaiza A, Goeman F, Benedetti A, Gallo E, Daralioti T, Perracchio L, Sacconi A, Pasanisi P, Muti P, Pulito C, Strano S, Ianniello Z, Fatica A, Forcato M, Fazi F, Blandino G, Fontemaggi G (2022) MALAT1-dependent hsa_circ_0076611 regulates translation rate in triple-negative breast cancer. Commun Biol. 10.1038/S42003-022-03539-X 35710947 10.1038/s42003-022-03539-x PMC9203778

[CR150] Verduci L, Tarcitano E, Strano S, Yarden Y, Blandino G (2021) Circrnas: role in human diseases and potential use as biomarkers. Cell Death Dis. 10.1038/S41419-021-03743-3 33976116 10.1038/s41419-021-03743-3 PMC8113373

[CR151] Voigtlaender M, Schneider-Merck T, Trepel M (2018) Lapatinib. Recent Results Cancer Res 211:19–44. 10.1007/978-3-319-91442-8_2 30069757 10.1007/978-3-319-91442-8_2

[CR152] Vromman M, Anckaert J, Bortoluzzi S, Buratin A, Chen CY, Chu Q, Chuang TJ, Dehghannasiri R, Dieterich C, Dong X, Flicek P, Gaffo E, Gu W, He C, Hoffmann S, Izuogu O, Jackson MS, Jakobi T, Lai EC, Volders PJ (2023) Large-scale benchmarking of circRNA detection tools reveals large differences in sensitivity but not in precision. Nat Methods 20(8):1159–1169. 10.1038/S41592-023-01944-6 37443337 10.1038/s41592-023-01944-6 PMC10870000

[CR153] Wang S, Liu F, Ma H, Cui X, Yang S, Qin R (2020) CircCDYL acts as a tumor suppressor in triple negative breast cancer by sponging miR-190a-3p and upregulating TP53INP1. Clin Breast Cancer 20(5):422–430. 10.1016/j.clbc.2020.04.006 32741666 10.1016/j.clbc.2020.04.006

[CR154] Wang X, Xue B, Zhang Y, Guo G, Duan X, Dou D (2021a) Up-regulated circBACH2 contributes to cell proliferation, invasion, and migration of triple-negative breast cancer. Cell Death Dis. 10.1038/S41419-021-03684-X 33875646 10.1038/s41419-021-03684-x PMC8055688

[CR155] Wang YW, Xu Y, Wang YY, Zhu J, Gao HD, Ma R, Zhang K (2021b) Elevated circrnas circ_0000745, circ_0001531 and circ_0001640 in human whole blood: potential novel diagnostic biomarkers for breast cancer. Exp Mol Pathol. 10.1016/j.yexmp.2021.104661 34139239 10.1016/j.yexmp.2021.104661

[CR156] Wang X, Chen T, Li C, Li W, Zhou X, Li Y, Luo D, Zhang N, Chen B, Wang L, Zhao W, Fu S, Yang Q (2022a) CircRNA-CREIT inhibits stress granule assembly and overcomes doxorubicin resistance in TNBC by destabilizing PKR. J Hematol Oncol 15(1):122. 10.1186/S13045-022-01345-W 36038948 10.1186/s13045-022-01345-w PMC9425971

[CR157] Wang X, Jian W, Luo Q, Fang L (2022b) CircSEMA4B inhibits the progression of breast cancer by encoding a novel protein SEMA4B-211aa and regulating AKT phosphorylation. Cell Death Dis. 10.1038/S41419-022-05246-1 36115854 10.1038/s41419-022-05246-1 PMC9482637

[CR158] Wang X, Song H, Fang L, Wu T (2022c) EIF4A3-mediated circprkci expression promotes triple-negative breast cancer progression by regulating WBP2 and PI3K/AKT signaling pathway. Cell Death Discov. 10.1038/S41420-022-00892-Y 35236829 10.1038/s41420-022-00892-y PMC8891274

[CR159] Wang S, Wang Y, Wang Y, Li Q, Zeng K, Li X, Feng X (2023a) Myc derived circrna promotes triple-negative breast cancer progression via reprogramming fatty acid metabolism. Discov Oncol. 10.1007/S12672-023-00679-2 37173608 10.1007/s12672-023-00679-2 PMC10182216

[CR160] Wang Z, Deng H, Jin Y, Luo M, Huang J, Wang J, Zhang K, Wang L, Zhou J (2023b) Circular rnas: biology and clinical significance of breast cancer. RNA Biol 20(1):859–874. 10.1080/15476286.2023.2272468 37882644 10.1080/15476286.2023.2272468 PMC10730165

[CR161] Wang D, Chen D, Liang L, Hu J (2024a) The circZEB1/miR-337–3p/OGT axis mediates angiogenesis and metastasis via o-GlcNAcylation and up-regulating YBX1 in breast cancer. Heliyon. 10.1016/j.heliyon.2024.e34079 39114035 10.1016/j.heliyon.2024.e34079 PMC11305230

[CR162] Wang D, Yang S, Lyu M, Xu L, Zhong S, Yu D (2024b) Circular RNA HSDL2 promotes breast cancer progression via miR-7978 ZNF704 axis and regulating hippo signaling pathway. Breast Cancer Res 26(1):105. 10.1186/S13058-024-01864-Z 38937788 10.1186/s13058-024-01864-z PMC11210124

[CR163] Wang S, Li Q, Wang Y, Li X, Feng X, Wei Y, Wang J, Zhou X (2024c) Peptidylprolyl isomerase D circular RNA sensitizes breast cancer to trastuzumab through remodeling HER2 N4-acetylcytidine modification. J Appl Genet. 10.1007/S13353-024-00840-9 38340287 10.1007/s13353-024-00840-9

[CR164] Wei J, Li M, Xue C, Chen S, Zheng L, Deng H, Tang F, Li G, Xiong W, Zeng Z, Zhou M (2023) Understanding the roles and regulation patterns of circRNA on its host gene in tumorigenesis and tumor progression. J Exp Clin Cancer Res. 10.1186/S13046-023-02657-6 37060016 10.1186/s13046-023-02657-6 PMC10105446

[CR165] Wu X, Ren Y, Yao R, Zhou L, Fan R (2021) Circular RNA circ-MMP11 contributes to lapatinib resistance of breast cancer cells by regulating the miR-153-3p/ANLN axis. Front Oncol. 10.3389/FONC.2021.639961 34295807 10.3389/fonc.2021.639961 PMC8290203

[CR166] Wu H, Wang A, Wang L, Shi F, Lin F, Cui H (2023) A novel circ_0104345/miR-876-3p/ZBTB20 axis regulates the proliferation, migration, invasion, and apoptosis of breast cancer cells. Biochem Genet 61(6):2548–2565. 10.1007/S10528-023-10391-Z 37148331 10.1007/s10528-023-10391-z

[CR167] Xiao J, Cohen IR, Lajtha A, Lambris JD, Paoletti R, Rezael N (2018) *Circular RNAs, Biogenesis and Functions* (Vol. 1724). 10.1007/978-981-13-1426-1

[CR168] Xiao W, Zheng S, Zou Y, Yang A, Xie X, Tang H, Xie X (2019) Circahnak1 inhibits proliferation and metastasis of triple-negative breast cancer by modulating miR-421 and RASA1. Aging 11(24):12043–12056. 10.18632/AGING.102539 31857500 10.18632/aging.102539 PMC6949091

[CR169] Xie H, Zheng R (2022) Circ_0085495 knockdown reduces adriamycin resistance in breast cancer through miR-873-5p/integrin β1 axis. Anticancer Drugs 33(1):E166–E177. 10.1097/CAD.0000000000001174 34387598 10.1097/CAD.0000000000001174

[CR170] Xing L, Yang R, Wang X, Zheng X, Yang X, Zhang L, Jiang R, Ren G, Chen J (2020) The circRNA circIFI30 promotes progression of triple-negative breast cancer and correlates with prognosis. Aging 12(11):10983–11003. 10.18632/AGING.103311 32497020 10.18632/aging.103311 PMC7346060

[CR171] Xing Z, Wang R, Wang X, Liu J, Zhang M, Feng K, Wang X (2021) CircRNA circ-PDCD11 promotes triple-negative breast cancer progression via enhancing aerobic glycolysis. Cell Death Discov. 10.1038/S41420-021-00604-Y 34420029 10.1038/s41420-021-00604-y PMC8380247

[CR172] Xu J, Lamouille S, Derynck R (2009) TGF-Β-induced epithelial to mesenchymal transition. Cell Res 19(2):156–172. 10.1038/CR.2009.5 19153598 10.1038/cr.2009.5 PMC4720263

[CR173] Xu JZ, Shao CC, Wang XJ, Zhao X, Chen JQ, Ouyang YX, Feng J, Zhang F, Huang WH, Ying Q, Chen CF, Wei XL, Dong HY, Zhang GJ, Chen M (2019) Circtada2as suppress breast cancer progression and metastasis via targeting miR-203a-3p/SOCS3 axis. Cell Death Dis. 10.1038/S41419-019-1382-Y 30787278 10.1038/s41419-019-1382-y PMC6382814

[CR174] Xu Y, Zhang S, Liao X, Li M, Chen S, Li X, Wu X, Yang M, Tang M, Hu Y, Li Z, Yu R, Huang M, Song L, Li J (2021) Circular RNA circikbkb promotes breast cancer bone metastasis through sustaining NF-κB/bone remodeling factors signaling. Mol Cancer. 10.1186/S12943-021-01394-8 34325714 10.1186/s12943-021-01394-8 PMC8320207

[CR175] Xu S, Luo W, Li M, Li Q, Hong W, Gao Y, Yang J, Song H, Chen li, Yang Y, Yang C (2023) Circ_0001667 promotes adriamycin resistance and malignant progression via targeting the miR-193a-5p/Rap2A molecular axis in breast cancer. Clin Breast Cancer 23(1):71–83. 10.1016/j.clbc.2022.09.008 36289041 10.1016/j.clbc.2022.09.008

[CR176] Xu R, Lan H, Zhang L, Yang S, Mao Y, Che H (2024) Circ_JMJD1C expedites breast cancer progression by regulating miR-182-5p/ JMJD1C/SOX4 axis. Cell Mol Biol 70(3):204–211. 10.14715/CMB/2024.70.3.31 38650133 10.14715/cmb/2024.70.3.31

[CR177] Yang L, Chen Y (2023) Circ_0008717 sponges miR-326 to elevate GATA6 expression to promote breast cancer tumorigenicity. Biochem Genet 61(2):578–596. 10.1007/S10528-022-10270-Z 36001185 10.1007/s10528-022-10270-z

[CR178] Yang S, Tang J (2020) Exosomal circular RNAs derived from serum: promising biomarkers for therapeutic targets and prognosis of triple-negative breast cancer (TNBC). J Clin Oncol 38(15suppl):3528–3528. 10.1200/JCO.2020.38.15_SUPPL.352832749942

[CR179] Yang Y-H, Mao J-W, Tan X-L (2020) Research progress on the source, production, and anti-cancer mechanisms of Paclitaxel. Chin J Nat Med 18(12):10–17. 10.1016/S1875-5364(20)60032-2 10.1016/S1875-5364(20)60032-2 33357719

[CR180] Yang C, Zhang S, Liu L, Zhang X, Du K, Gao C, Li J, Liu Y (2023) Hsa_circ_0002496 promotes the growth, angiogenesis, and stemness of breast cancer cells via miR-433-3p/YWHAZ cascade. Thorac Cancer 14(18):1732–1741. 10.1111/1759-7714.14918 37160403 10.1111/1759-7714.14918 PMC10290916

[CR181] Yang B, Wang YW, Zhang K (2024) Interactions between circRNA and protein in breast cancer. Gene. 10.1016/j.gene.2023.148019 37984538 10.1016/j.gene.2023.148019

[CR182] Yao J, Deng K, Huang J, Zeng R, Zuo J (2020) Progress in the understanding of the mechanism of Tamoxifen resistance in breast cancer. Front Pharmacol. 10.3389/FPHAR.2020.592912 33362547 10.3389/fphar.2020.592912 PMC7758911

[CR183] Yarmishyn AA, Ishola AA, Chen CY, Verusingam ND, Rengganaten V, Mustapha HA, Chuang HK, Teng YC, Long P, Van, Hsu PK, Lin WC, Ma HI, Chiou SH, Wang ML (2022) Circular RNAs modulate cancer hallmark and molecular pathways to support cancer progression and metastasis. Cancers 14(4). 10.3390/CANCERS14040862 10.3390/cancers14040862 PMC8869994 35205610

[CR184] Ye F, Gao G, Zou Y, Zheng S, Zhang L, Ou X, Xie X, Tang H (2019) Circfbxw7 inhibits malignant progression by sponging miR-197-3p and encoding a 185-aa protein in Triple-Negative breast cancer. Mol Ther Nucleic Acids 18:88–98. 10.1016/j.omtn.2019.07.023 31536884 10.1016/j.omtn.2019.07.023 PMC6796723

[CR185] Yi J, Du J, Chen X, Nie RC, Hu GS, Wang L, Zhang YY, Chen S, Wen XS, Luo DX, He H, Liu W (2025) A circRNA–mRNA pairing mechanism regulates tumor growth and endocrine therapy resistance in ER-positive breast cancer. Proc Natl Acad Sci U S A. 10.1073/PNAS.2420383122 40233410 10.1073/pnas.2420383122 PMC11874584

[CR186] Yin WB, Yan MG, Fang X, Guo JJ, Xiong W, Zhang RP (2018) Circulating circular RNA hsa_circ_0001785 acts as a diagnostic biomarker for breast cancer detection. Clin Chim Acta 487:363–368. 10.1016/j.cca.2017.10.011 29045858 10.1016/j.cca.2017.10.011

[CR187] Youness RA, Hassan HA, Abaza T, Hady AA, Magdoub E, Ali HM, Vogel M, Thiersch J, Gassmann M, Hamdy M, N. M., Aboouf MA (2024) A comprehensive insight and in Silico analysis of circrnas in hepatocellular carcinoma: A step toward ncRNA-Based precision medicine. Cells 13(15). 10.3390/CELLS13151245 10.3390/cells13151245 PMC11312109 39120276

[CR188] Yu J, Shen W, Xu J, Gong B, Gao B, Zhu J (2020) CircUSP42 is downregulated in Triple-Negative breast cancer and associated with poor prognosis. Technol Cancer Res Treat. 10.1177/1533033820950827 32938310 10.1177/1533033820950827 PMC7502800

[CR189] Yuan M, Zhang J, He Y, Yi G, Rong L, Zheng L, Zhan T, Zhou C (2022) Circ_0062558 promotes growth, migration, and glutamine metabolism in triple-negative breast cancer by targeting the miR-876-3p/SLC1A5 axis. Arch Gynecol Obstet 306(5):1643–1655. 10.1007/S00404-022-06481-9 35284960 10.1007/s00404-022-06481-9

[CR190] Zang J, Lu D, Xu A (2020) The interaction of circrnas and RNA binding proteins: an important part of circrna maintenance and function. J Neurosci Res 98(1):87–97. 10.1002/JNR.24356 30575990 10.1002/jnr.24356

[CR191] Zeng Y, Zou Y, Gao G, Zheng S, Wu S, Xie X, Tang H (2022) The biogenesis, function and clinical significance of circular RNAs in breast cancer. Cancer Biol Med 19(1):14–29. 10.20892/J.ISSN.2095-3941.2020.0485 10.20892/j.issn.2095-3941.2020.0485 PMC8763001 34110722

[CR192] Zepeda-Enríquez P, Silva-Cázares MB, López-Camarillo C (2023) Novel insights into circular RNAs in metastasis in breast cancer: an update. Non-Coding RNA 9(5):55. 10.3390/NCRNA9050055 37736901 10.3390/ncrna9050055 PMC10514845

[CR193] Zhang Y, Zhang XO, Chen T, Xiang JF, Yin QF, Xing YH, Zhu S, Yang L, Chen LL (2013) Circular intronic long noncoding RNAs. Mol Cell 51(6):792–806. 10.1016/j.molcel.2013.08.017 24035497 10.1016/j.molcel.2013.08.017

[CR194] Zhang H, da, Jiang L, hong, Sun D, wei, Hou Jchen, Ji Z (2018) ling. CircRNA: a novel type of biomarker for cancer. Breast Cancer, 25(1). 10.1007/S12282-017-0793-9 10.1007/s12282-017-0793-9 28721656

[CR195] Zhang Y, Nguyen TM, Zhang XO, Wang L, Phan T, Clohessy JG, Pandolfi PP (2021) Optimized RNA-targeting CRISPR/Cas13d technology outperforms ShRNA in identifying functional circrnas. Genome Biol. 10.1186/S13059-021-02263-9 33478577 10.1186/s13059-021-02263-9 PMC7818937

[CR196] Zhang Y, Tan D, Xie Y, Wu L, Wu S, Tang Y, Luo Y, Xiao X, Li X (2022) Circepsti1 promotes the proliferation of HER2-Positive breast cancer cells via circEPSTI1/miR-145/ERBB3 axis. J Oncol. 10.1155/2022/1028851 36059813 10.1155/2022/1028851 PMC9439903

[CR197] Zhang C, Wang J, Wang H, Li J (2024) Interference of the circular RNA sperm antigen with Calponin homology and Coiled-Coil domains 1 suppresses growth and promotes apoptosis of breast cancer cells partially through targeting miR-1236-3p/Chromobox 8 pathway. Clin Breast Cancer 24(3):e138–e151e2. 10.1016/j.clbc.2023.11.009 38341369 10.1016/j.clbc.2023.11.009

[CR198] Zhao Y, Wang H (2025) Artificial intelligence-driven circRNA vaccine development: multimodal collaborative optimization and a new paradigm for biomedical applications. Brief Bioinform. 10.1093/BIB/BBAF263 40483546 10.1093/bib/bbaf263 PMC12145227

[CR199] Zhong W, Bao L, Yuan Y, Meng Y (2021) Circrassf2 acts as a prognostic factor and promotes breast cancer progression by modulating miR-1205/HOXA1 axis. Bioengineered 12(1):3014–3028. 10.1080/21655979.2021.1933300 34180753 10.1080/21655979.2021.1933300 PMC8806576

[CR200] Zhong S, Xu H, Wang D, Yang S, Li H, Zhang H, Feng J, Zhou S (2024) Circnfib decreases synthesis of arachidonic acid and inhibits breast tumor growth and metastasis. Eur J Pharmacol. 10.1016/j.ejphar.2023.176221 38128869 10.1016/j.ejphar.2023.176221

[CR201] Zhou J, Zhang WW, Peng F, Sun JY, He ZY, Wu SG (2018) Downregulation of hsa_circ_0011946 suppresses the migration and invasion of the breast cancer cell line MCF-7 by targeting RFC3. Cancer Manage Res 10:535–544. 10.2147/CMAR.S155923 10.2147/CMAR.S155923 PMC5865555 29593432

[CR202] Zhou WY, Cai ZR, Liu J, Wang DS, Ju HQ, Xu RH (2020a) Circular RNA: metabolism, functions and interactions with proteins. Mol Cancer 19(1):172. 10.1186/S12943-020-01286-3 33317550 10.1186/s12943-020-01286-3 PMC7734744

[CR203] Zhou Y, Liu X, Lan J, Wan Y, Zhu X (2020b) Circular RNA circRPPH1 promotes triple-negative breast cancer progression via the miR-556-5p/YAP1 axis. Am J Transl Res 12(10):6220 33194025 PMC7653573

[CR204] Zhu Z, Yu Z, Rong Z, Luo Z, Zhang J, Qiu Z, Huang C (2019) The novel GINS4 axis promotes gastric cancer growth and progression by activating Rac1 and CDC42. Theranostics 9(26):8294–8311. 10.7150/THNO.36256 31754397 10.7150/thno.36256 PMC6857050

[CR205] Zhu K, Yi C, Tong C (2024) Circ_0058063 promotes breast cancer progression by upregulating DLGAP5 via sponging miR-557. Cancer Biomark 39(1):1–13. 10.3233/CBM-220410 37334578 10.3233/CBM-220410 PMC10977444

[CR206] Zhuang M, Zhang X, Ji J, Zhang H, Shen L, Zhu Y, Liu X (2024) Exosomal circ-0100519 promotes breast cancer progression via inducing M2 macrophage polarisation by USP7/NRF2 axis. Clin Transl Med 14(8):e1763. 10.1002/CTM2.1763 39107958 10.1002/ctm2.1763 PMC11303452

